# Total Synthesis and Antimalarial Studies of Caelestines A–C

**DOI:** 10.1002/cmdc.70345

**Published:** 2026-06-15

**Authors:** Henry S. T. Smith, Aaron M. Lock, Vicky M. Avery, Rohan A. Davis

**Affiliations:** ^1^ Institute for Biomedicine and Glycomics Griffith University Nathan Queensland Australia; ^2^ School of Environment and Science Griffith University Nathan Queensland Australia; ^3^ Discovery Biology Griffith University Nathan Queensland Australia; ^4^ NatureBank Griffith University Nathan Queensland Australia

**Keywords:** antimalarial natural product, caelestine, quinolone, structure–activity relationship, total synthesis

## Abstract

Caelestines A–D are 2‐carboxy‐4‐quinolones (2C4Qs) first isolated from the Australian ascidian *Aplidium caelestis*. The caelestines are brominated analogues of kynurenic acid and xanthurenic acid, and the only brominated 2C4Qs isolated from nature. A convenient methodology exists for the Conrad synthesis of 2C4Qs from substituted anilines and acetylene dicarboxylates cyclised in Eaton's reagent. This approach yields regioisomers from asymmetric anilines and is hampered by the absence of chromatographic methods for isolation of pure isomers. Here, we report de novo synthesis of caelestines A–C using this methodology, constituting the first reported syntheses of caelestines B and C, as well as the first reported synthesis of five other novel bromo‐quinolones and three related intermediates. The first chromatographic separation of caelestine isomers yielded by Conrad synthesis is described, and structure–activity relationships of caelestines A–C (and related bromo‐quinolones) against the *Plasmodium falciparum* 3D7 (chloroquine‐sensitive) and Dd2 (chloroquine‐resistant) strains are elucidated.

## Graphical abstract

Total synthesis of caelestines A–C (alkaloids from the Australian ascidian, *Aplidium caelestis*) was accomplished, constituting the first synthesis of caelestines B and C. Caelestines A–C, with five other novel quinolones, were assessed against *Plasmodium falciparum* 3D7 and Dd2. Caelestine B was found to be a selective antimalarial alkaloid.

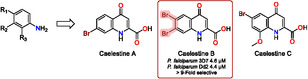

## 1 Introduction

**FIGURE 1:**
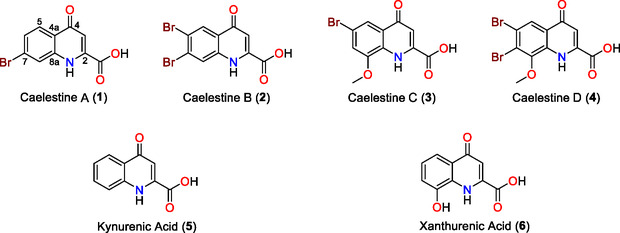
The structures of the caelestines A−D (**1**–**4**) and the kynurenine metabolites, kynurenic acid (**5**) and xanthurenic acid (**6**).

The caelestines A–D (**1**–**4**) are brominated 2‐carboxy‐4‐quinolones (2C4Qs; Figure 1) originally isolated from a sample of the Australian ascidian *Aplidium caelestis* [1]. These compounds were the first brominated quinoline carboxylic acid scaffolds reported to have been isolated from nature [1] and remain the only such natural products reported to date [2]. Biosynthesis of the quinoline scaffold is presumed to occur as a result of kynurenine metabolism (catabolism of tryptophan), because the caelestines are brominated derivatives of the kynurenine pathway products, kynurenic acid (**5**) and xanthurenic acid (**6**; Figure 1) [3]. Brominated metabolites and brominating enzymes are widely described in certain marine micro‐organisms [4], and the occurrence of brominated 2C4Qs in *Aplidium caelestis* most likely occurs by utilisation of ultimately exogenous, bromotryptophan‐derived substrates.

At first isolation [1], caelestines A–D (**1**–**4**) were assessed for antiproliferative activity against the breast adenocarcinoma cell line MCF‐7, the melanoma cell line MM96L, and non‐malignant neonatal foreskin fibroblasts (NFF) [1]. Non‐selective cytotoxic activity (38%–69% growth inhibition) was observed against these cell lines at the assay top‐dose of 100 µM [1]. The only other investigation of the biological activity of **1**–**4** reported that caelestine C (**3**), and its kynurenine analogue xanthurenic acid (**6**), were inhibitors of U87‐MG and U251‐MG glioblastoma transwell invasion [5]. The 2C4Q scaffold is a known synthetic class, but of the caelestines A−D, only caelestine A has ever been synthesised in studies prior to [6, 7] or following [8] the original isolation of the caelestines [2]. As a brominated derivative of the neuroprotective metabolite kynurenic acid, caelestine A has been investigated by in vitro and in silico assays for *N*‐methyl‐D‐aspartate (NMDA) receptor antagonism, and functioned as an antagonist [7, 9, 10].

**SCHEME 1:**
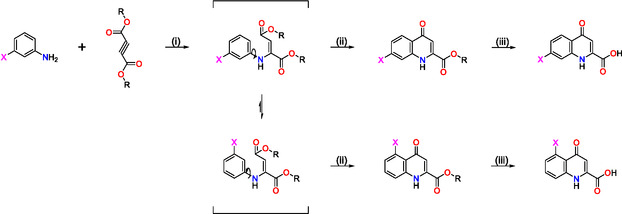
Generic scheme describing Conrad synthesis of 2C4Qs starting with asymmetric aniline. A pair of regioisomers results from rotation about the aniline bond. (i) R‐OH, rt, 3 h. (ii) Eaton's reagent, 50°C, 2 h. (iii) Base (aq), rt, 2 h.

The 2C4Q scaffold is synthetically accessible by Biere–Seelen [11] substitution of acetylene dicarboxylates with commercially available anilines at room temperature (rt), and cyclisation of the resulting enamine at low temperatures in Eaton's reagent (phosphorous pentoxide in methanesulfonic acid), followed by alkali hydrolysis of the resulting carboxylate (Scheme 1) [12]. This modified Conrad quinolone synthesis using Eaton's reagent was first reported by Merck scientists in 2007 and Eaton's reagent provides some catalytic or pseudocatalytic species, which allows electrocyclisation to occur at very low temperature compared to traditional Conrad synthesis (50°C vs. 250°C) [12]. When the starting aniline bears asymmetrical substitution and two enamine carbon, Conrad synthesis yields a regioisomer along with the intended product, following cyclisation of the enamine intermediate [12]. This regioisomeric pair of products is presumably a result of the enamine intermediate existing as a pair of pseudostable rotamers about the aniline bond, with each rotamer yielding a different regioisomer (Scheme 1).

The starting anilines required for both caelestines A and B are asymmetric and yield regioisomeric pairs after Conrad synthesis [12], but for these studies, the bromination pattern appeared important to structure–activity relationships (SAR) within the series, and these regioisomers were deemed desirable for acquiring SAR data. Thus, modified Conrad synthesis was employed for the de novo synthesis of the caelestines A–C, constituting the first report of the synthesis of caelestines B and C, along with five other novel quinolones and three novel enamines. Ultimately, the caelestines A–C, together with related quinolones and intermediates in the synthesis, were obtained in high purity (≥95%). Caelestines A–C were tested against the non‐tumorigenic cell‐line HEK293, in order to make a preliminary assessment of compound toxicity. The entire panel of quinolones (**1**–**3**, **10**–**16**) was then tested against the chloroquine‐sensitive *Plasmodium falciparum (Pf)* 3D7, and the chloroquine‐resistant *Pf*Dd2 malaria stain, to furnish SAR data for the compounds and assess the compounds’ potential as anti‐infective agents.

## 2 Results and Discussion

### 2.1 Total Synthesis of Caelestines A–C

**SCHEME 2:**
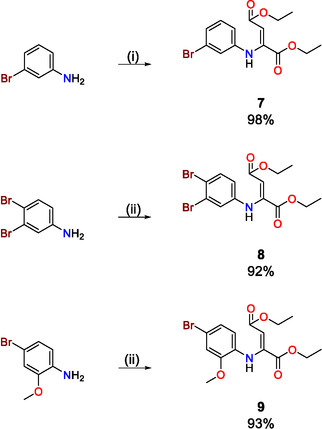
Synthesis of enamine precursors (**7**–**9)** of caelestines A–C. (i) DEAD, Ar, 0°C, 20 min; 50°C 3 h. (ii) DEAD, EtOH, Ar, 0°C, 20 min; 50°C, 3 h.

Studies began with the synthesis of the novel carboxylate enamine precursors **7**–**9** [2], using the relevant commercially available anilines and diethylacetylene dicarboxylate (DEAD), consistent with previously reported protocols (Scheme 2) [12]. For each enamine, ice cold DEAD was added to starting aniline over an ice bath and stirred for 20 min, before gently heating to 50°C, followed by stirring for a further 3 h, under argon atmosphere. The starting aniline required for the synthesis of caelestine D precursor using this method, 3,4‐dibromo‐2‐methoxyaniline, is not widely commercially available and a convenient synthesis has not been described [2], so the synthesis of caelestine D was not attempted here. The enamines **7** and **8** refused to precipitate as recoverable solid, even after intensive in vacuo drying. The enamine **9** was obtained after drying as a waxy off‐white solid. All enamines could be precipitated as white waxy solids by addition of a drop of H_2_O to the reaction mixtures, but filtration of the solid was complicated by the enamines’ high solubility in organics, and the need to remove H_2_O before cyclisation. It was deemed preferable to avoid heating filtered enamine solid in the presence of H_2_O, so instead of filtration and oven drying, enamine products were extracted in CH_2_Cl_2_ from 1.0 M HCl (aq) and then freeze‐dried. Negligible H_2_O or CH_2_Cl_2_ were detected in the resulting enamine gums by ^1^H NMR spectroscopy (Supporting Information, S14, S17, and S20) and using this work‐up, pure enamines **7**–**9** were obtained in 98%, 92% and 93% yield, respectively.

**SCHEME 3:**
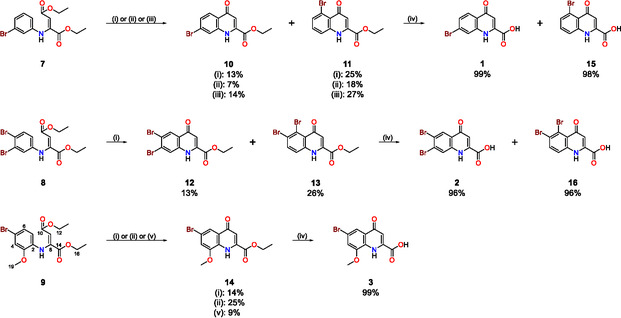
Electrocyclisation of enamines **7**–**9** in Eaton's reagent at different temperatures. (i) Eaton's reagent, Ar, 50°C, 2 h. (ii) Eaton's reagent, Ar, 90°C, 2 h. (iii) Eaton's reagent, Ar, rt, 2 h. (iv)10% TEA/H_2_O v/v with MeCN, 50°C, 2 h. (v) Eaton's reagent, Ar, 70°C, 2 h.

These enamines **7**–**9** were then cyclised in Eaton's reagent according to Scheme 3. Reaction mixtures were neutralised using 1.0 M Na_2_CO_3_ (aq) to yield yellow solids which were vacuum filtered and washed sparingly with ice cold acetone, giving white solids consisting of mixed quinolone isomers from **7** and **8** respectively, and semipure **14** from enamine **9**. The quinolone ethyl carboxylates **10**–**14** were ultimately obtained in high purity (≥95%, assessed by ^1^H and ^13^C NMR and UHPLC‐MS), following development of an effective chromatographic purification (discussed below). In each applicable case, the less desirable regioisomer was favoured in roughly 2:1 proportion compared to the ethyl carboxylate of the natural product (Scheme 3). To investigate whether the distribution of these isomers could be equalised at higher temperature, the cyclisation of **7** was conducted at 90°C but this resulted in decreased yields (Scheme 3). To investigate whether the yield or product distribution could be altered at lower temperature, the cyclisation of **7** was then conducted at rt, but this made no substantial difference in overall yield or the relative yields of regioisomers **10** and **11**. Electrocyclisation of enamine **9** to the quinolone **14** gave lower overall yield compared to **10**–**13** when cyclisation was conducted at 50°C. It was suspected that this comparatively low yield was caused by a reduced rate of conversion, owing to obstruction of an otherwise available β‐position by the methoxy substituent at position‐3 of enamine **9** **(**Scheme 3). The reaction was then conducted at 70°C, rather than extend the reaction time in harsh Eaton's reagent, and the yield of **14** was subsequently improved from 14% to 25%. The same reaction was subsequently attempted at 90°C, but in a manner similar to **7**, cyclisation of **9** in Eaton's reagent at 90°C lowered the overall yield (9%).

Yields of the ethyl carboxylates **10**–**14** (of which all but **10** were novel) [2] were serviceable (13%–26%), and conveniently, alkali hydrolysis to the target carboxylic acids (**1**, **2**, **15**, and the novel quinolone **16**) with 1.0 M NaOH (aq) was rapid (92%–98% recovery). However, the use of 1.0 M NaOH resulted in a great deal of excess salt, which was an impediment to dissolving the resulting crude mixture for liquid injection onto a HPLC system. A more convenient hydrolysis using 10% triethylamine (TEA) in H_2_O (v/v), with a few drops acetonitrile (MeCN), also completely hydrolysed the ethyl carboxylates **10**–**14** (96%–99% recovery) with gentle heating. Previously reported syntheses of caelestine A involved repeated recrystallisation of the slightly less soluble caelestine A from its 5‐bromo isomer. To be certain of isomer purity, a RP‐HPLC chromatography method for the separation of bromokynurenic acids at semipreparative scale was developed for these studies (Supporting Information, S44). Ultimately, a Waters LC Prep AutoPurification System was used for separations and all quinolones were obtained in high purity (≥95%). This approach gave caelestines A–C (**1**–**3**) in 13%, 12% and 23% overall yield, respectively.

Moderate to severe solubility issues were observed for all pure quinolones, both carboxylates and carboxylic acids, including the caelestines A–C (**1**–**3**), but these issues did not preclude unambiguous 2D NMR assignments (^1^H, ^13^C, COSY, HSQC, HMBC) for all quinolones and enamines (Supporting Information, S1–S42). The quinolones proved most soluble in DMSO‐D_6_ and DMF‐D_7_ and were marginally more soluble in DMF‐D_7_, so DMF‐D_7_ was used for all NMR experiments. The ^1^H and ^13^C NMR spectra for the synthesised caelestines A–C (**1**–**3**; in MeOH‐D_4_) were compared to previously published spectra [1] for the caelestines A–C in MeOH‐D_4_ and were similarly compared directly to retained authentic reference samples for caelestines A–C (isolated from *Aplidium caelestis*) and found to be identical (Supporting Information, S3, S7, and S11).

### 2.2 Antimalarial Studies

**FIGURE 2:**
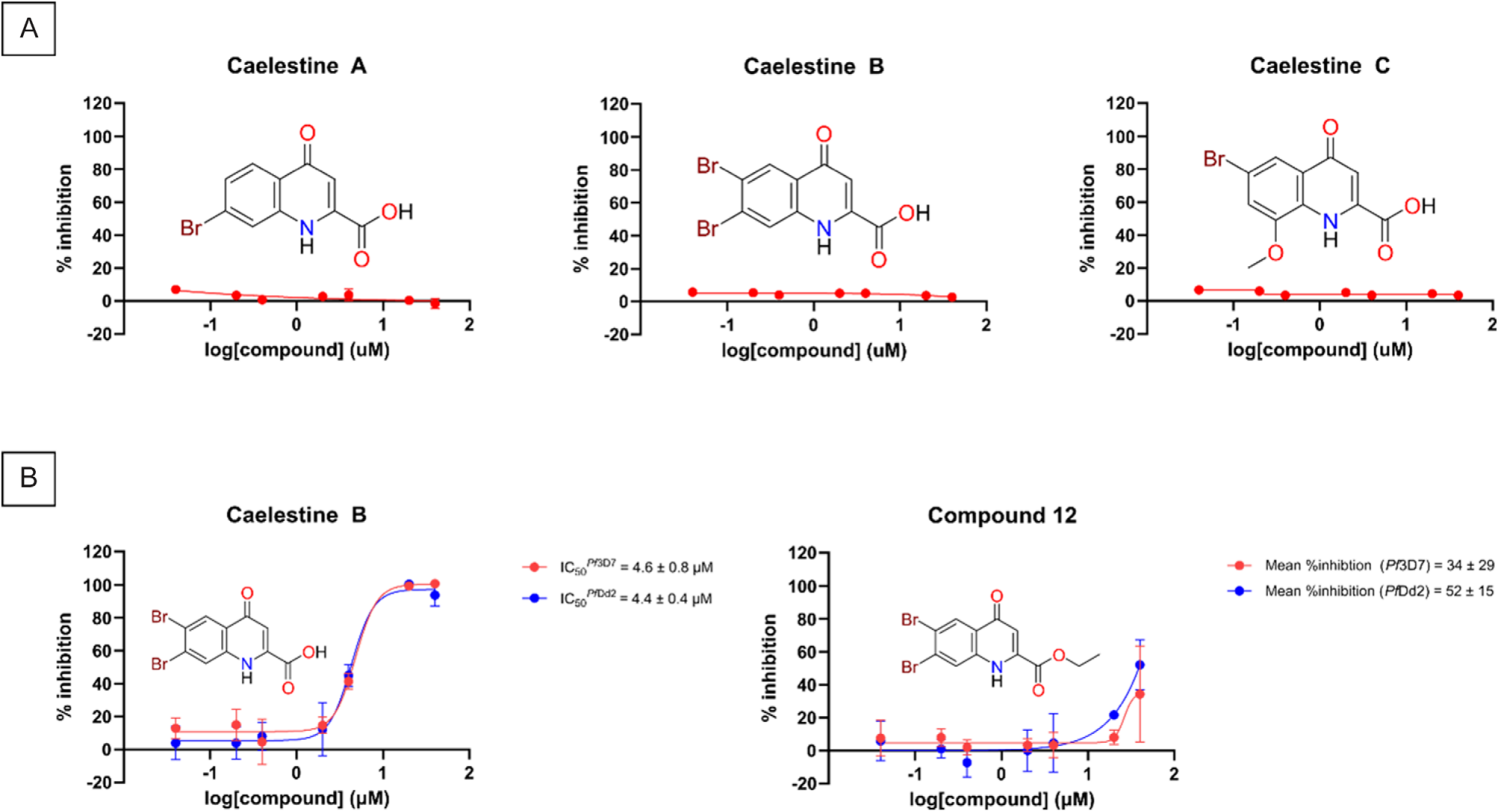
Selected dose–response curves for biological studies of the quinolone compound panel (**1**–**3** and **16**). (A) Dose‐response curves for caelestines A–C (**1**–**3**) against HEK293 (*N* = 1, triplicate point). (B) Dose‐response curves for caelestine B (**2**) and its carboxylate isomer **12,** against *P. falciparum* 3D7 and Dd2 (*N* = 3, single point). All error bars represent standard deviation.

The caelestines A–C (**1**–**3**) were previously evaluated against neonatal foreskin fibroblasts (NFF) and found to exert non‐selective growth inhibition (38%–69%) at the assay top‐dose of 80 µM with 96 h treatment. Owing to the very high purity of **1**–**3** synthesised in these studies, **1**–**3** were reassessed for cytotoxicity against the non‐tumorigenic cell line HEK293 in a resazurin‐based assay [13]. No growth inhibition of HEK293 was observed for **1**–**3** following 72 h treatment at 40 µM (Figure 2), the highest concentration tested. All quinolones (**1**–**3** and **10**–**16**) were then tested against *P. falciparum* 3D7 (chloroquine‐sensitive) and Dd2 (chloroquine‐resistant) strains in a robust and well‐validated image‐based in vitro protocol [14], in order to furnish SAR data.

Interpretation of SAR trends for the quinolones tested was greatly simplified by wide‐spread inactivity against *Pf*3D7 and *Pf*Dd2. Only caelestine B (**2**) proved active in the screen, while some inhibition at the assay top‐dose was observed for the ethyl carboxylate of caelestine B, quinolone **12**. Caelestine B showed clear sigmoidal kinetics characteristic of cidal activity, with IC_50_ values of 4.6 ± 0.8 µM (*Pf*3D7) and 4.4 ± 0.4 µM (*Pf*Dd2). Caelestine B was therefore >9‐fold selective for both *Pf*3D7 and *Pf*Dd2 compared to HEK293. The loss of activity for the ethyl carboxylate analogue **12** compared to caelestine B (**2**), also suggests that dibromination did not render **2** active by increasing lipophilicity and cell penetrance. Both caelestine B (**2**) and **12** are 6,7‐dibromokynurenic acids but the 7‐bromokynurenic acid, caelestine A (**1**), was either 9‐fold less active or entirely inactive compared to **2**, demonstrating that 7‐bromination was insufficient to confer antimalarial activity. Likewise, the 5,6‐dibromokynurenic acid **16** was not active in these studies. These data suggest that 6,7‐dibromination is a specific substitution pattern required for a >9‐fold increase in antimalarial activity for simple 2C4Qs (kynurenic acids), or is required for any antimalarial activity to be exerted at all.

**FIGURE 3:**
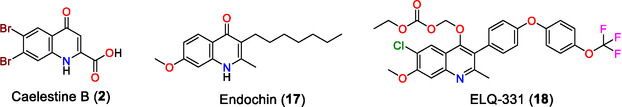
The structures of caelestine B (**2**), and the electron transport chain inhibitor, endochin, along with the advanced antiplasmodial ELQ candidate, ELQ‐331.

*P. falciparum* Dd2 is a long‐employed model for identifying compounds for which chloroquine resistance is indicated, and is also resistant (to various extents) to several other quinoline‐based antimalarials, such as mefloquine and quinine/quinidine [15]. Virtually identical IC_50_ values obtained for caelestine B (**2**) against both *Pf*3D7 and *Pf*Dd2 (4.6 and 4.4 µM) indicate that caelestine B was not rendered inactive by *Pf*Dd2 quinoline resistance mechanisms or drug efflux. These data also do not exclude the possibility that caelestine B exerts antimalarial activity by some different mechanism than the haemozoin‐crystal inhibition mechanism exerted by clinical antimalarial quinolines [16]. Endochin‐like quinolones (ELQs; Figure 3) are 4‐quinolone derivatives of the legacy synthetic endochin [17], and have elicited renewed interest in the 21^st^ century as electron transport chain (*bc*_1_ complex) inhibitors [18, 19] having been shown to possess selective activity against several protozoan pathogens, including *Plasmodium falciparum* [17, 19, 20]. It is notable that caelestine B bears a certain resemblance to ELQs and could likewise function as an electron transport chain inhibitor, especially in light of the fact that chlorination at position‐6 with concomitant methoxylation at position‐7 has been described as an effective strategy for improving antimalarial activity of ELQs and for overcoming atovaquone cross‐resistance [16]. This substitution pattern is somewhat comparable to 6,7‐dibromination. It is otherwise striking that 6,7‐dibromination is all that is required to convert the native human serum metabolite and ubiquitous tryptophan catabolite, kynurenic acid, into an antimalarial compound effective against a quinoline‐resistant strain, in vitro.

## 3 Conclusion

The caelestines A–C (**1**–**3**) were synthesised together with related quinolones (**10**–**16**), constituting the first report of the synthesis of **2**, **3**, **7**–**9**, **11**–**14**, and **16**. In both cases where regiosomeric mixtures of ethyl carboxylate precursors were yielded after electrocyclisation (**10**, **11** and **12**, **13**), the 5‐bromoisomer was favoured in a 2:1 ratio, which could not be shifted by altering reaction temperature. Chromatographic separation of the isomeric mixtures yielded by the modified Conrad synthesis was investigated and achieved but proved challenging and required specialist instrumentation at a semipreparative scale. Ultimately, all target quinolones were obtained in high purity (≥95%) via chromatographic methods (assessed by ^1^H and ^13^C NMR and UHPLC‐MS). The synthesised natural products **1**–**3** were assayed against non‐tumorigenic HEK293 cells, and no growth inhibition was observed at 40 µM (the highest concentration tested) at 72 h. All quinolones were assayed against *P. falciparum* 3D7 and Dd2 strains. Only caelestine B (**2**) showed pronounced activity, with IC_50_ values of 4.6 µM and 4.4 µM against both *Pf*3D7 and *Pf*Dd2, respectively. Caelestine B also showed good selectivity, and was >9‐fold selective for *Pf*3D7 and *Pf*Dd2 compared with HEK293. The SAR data obtained from the biological assay indicated that 6,7‐dibromination was a requirement for good antimalarial activity amongst the quinolone library tested. A similar pattern of substitution has been reported to result in increased activity against *Plasmodium* parasites for ELQs [16], and caelestine B may function in a manner similar to ELQ antimalarials.

## 4 Experimental Section

### 4.1 General Experimental

NMR spectra were recorded at 25°C on a Bruker (Billerica, MA, USA) AVANCE III HD 800 MHz NMR spectrometer equipped with a cryoprobe. MestreNova version 14.3.3 software was used for NMR data analysis. The ^1^H and ^13^C NMR chemical shifts were referenced to solvent peaks for DMF‐D_7_ (δ_H_ 8.03, δ_C_ 163.15) and DMSO‐D_6_ (δ_H_ 2.50, δ_C_ 39.52), or were referenced to solvent peaks for MeOD (δ_H_ 3.31, δ_C_ 49.00). HRESIMS data were acquired on a Bruker maXis II ETD ESI‐qTOF. LRESIMS data were recorded on a Thermo Fisher Scientific (Waltham, MA, USA) UltiMate 3000 RS UHPLC with a Thermo Fisher Scientific Accucore C_18_ column (2.6 μm, 80 Å, 150 × 2.1 mm) and UltiMate 3000 VW UV detector and Thermo Scientific ISQ EC single quadrupole ESI mass spectrometer. Measurement of chromatography solution pH was made using a TPS (Brisbane, Qld, Australia) 1852 mV pH metre. Alltech (Lexington, KY, USA) Davisil C_18_‐bonded silica (35–70 µm, 60 Å) were used for pre‐adsorption work before reversed‐phase HPLC separations. Pre‐absorbed material was packed into an Alltech stainless steel guard cartridge (10 × 30 mm) then attached to an HPLC column prior to separation. A Thermo Fisher Scientific BetaSil C_18_‐bonded silica (5 μm, 100 Å, 150 × 21.2 mm) column was used for reversed‐phase HPLC separations, as were an XBridge BEH C_18_‐bonded column (5 μm, 130 Å, 150 × 21.2 mm) and a Thermo Fisher Scientific BetaSil phenyl‐bonded silica (5 μm, 100 Å, 250 × 21.2 mm) column. Waters LC Prep AutoPurification System isolations were performed using a Waters 3767 autoinjector/fraction collector liquid handling system coupled to a Waters XBridge BEH C_18_‐bonded column (5 μm, 130 Å, 150 × 21.2 mm), analytes were detected by an ACQUITY QDa II Mass Detector (in positive mode) and a Waters 2487 Dual Wavelength Absorbance Detector; fraction collection was triggered based on UV absorbance at 254 nm. All compounds isolated with a Waters LC Prep AutoPurification System were purified using a linear gradient from 10% MeOH (0.1% TFA)/H_2_O (0.1% TFA) to 100% MeOH (0.1% TFA) over 18 min, with a constant flow rate of 20 mL/min. All solvents used for chromatography and MS were Honeywell Burdick & Jackson (Muskegon, MI, USA) or RCI Labscan (Bangkok, Thailand) HPLC grade. H_2_O was filtered using a Sartorius (Gottingen, Lower Saxony, Germany) Arium Pro VF ultrapure water system. Synthetic reagents were purchased from Sigma‐Aldrich (St. Louis, MO, USA) and used without further purification.

### 4.2 In Vitro Antiplasmodial Image‐Based Assay

*Plasmodium falciparum* 3D7 (MRA‐102; BEI Resources ATCC; Manassas, VA, USA) and Dd2 (MRA‐150; BEI Resources) were cultured in RPMI 1640 (Sigma‐Aldrich) supplemented with 2.5 mg/mL Albumax II (Life Technologies, Camarillo, CA, USA), 5% AB human serum (Sigma‐Aldrich), 25 mM HEPES (Sigma‐Aldrich), and 0.37 mM hypoxanthine (Sigma‐Aldrich). Human red blood cells (O+) were supplied by Australian Red Cross LifeBlood in accordance with agreement 23‐05QLD‐23. Use of human RBC for antiplasmodial experimentation was in accordance with Griffith University Human Ethics Exemption Approval #03/08/11019. Ring stage parasites were treated with compounds following two rounds of sorbitol synchronisation, as previously described [14]. Following incubation of assay plates (384‐well PhenoPlate, Revvity, Waltham, MA, USA) for 72 h at 37°C, and 5% CO_2_ and 5% O_2_, parasites were stained with 2‐(4‐amidinophenyl)‐1*H*‐indole‐6‐carboxamidine (DAPI; Sigma‐Aldrich) and imaged using an Opera Phenix High Content Screening System (Revvity). Images were analysed using Harmony software v.4.8 (Revvity). Experiments were performed as three independent experiments in single point (*N* = 3, single point). Puromycin dihydrochloride (Sigma‐Aldrich), chloroquine (Sigma‐Aldrich), and dihydroartemisinin (Sigma‐Aldrich) were used as in‐plate reference compounds. Reference compounds were used as in‐plate controls (*N* = 3, duplicate point) for all assay plates, and the data presented for reference compounds is indicative of six replicates each for *Pf*3D7 and *Pf*Dd2 (Supporting Information, S43).

### 4.3 In Vitro Cytotoxicity Assay

Human embryonic kidney cells, HEK293 (CRL‐1573; ATCC; Manassas, VA, USA), were maintained in DMEM (Life Technologies) containing 10% FBS (Bovogen Biologicals, East Keilor, VIC, Australia). Cytotoxicity testing was undertaken with minor modifications to a previously described protocol [13]. In brief, 5 μL of test compound solution was added to the wells of black/clear tissue culture‐treated, 384‐well plates (Greiner Bio‐One, Frickenhausen, Germany) containing 2000 adherent HEK293 cells/well in 45 μL and the plates were incubated for 66 h at 37°C in 5% CO_2_. After incubation, 5 μL of 600 μM resazurin (Cayman Chemical, Ann Arbor, MI, USA), diluted in growth media, was added to each well. Plates were incubated for an additional 6 h and measured for fluorescence at 535 nm excitation and 595 nm emission using an EnSpire plate reader. The % inhibition was calculated using 0.4% v/v DMSO (no inhibition) and 40 μM puromycin dihydrochloride (Sigma‐Aldrich) data (100% inhibition). IC_50_ values were obtained by plotting % inhibition against log dose by using GraphPad Prism v.10.0.2 (Dotmatics, San Diego, CA, USA) and using non‐linear regression with a variable slope plot. Puromycin was included as a reference compound to assess assay validity. Experiments were performed as *N* = 1, triplicate point. Reference compound (puromycin; Supporting Information, S43) served as an in‐plate control (*N* = 1, triplicate point).

### 4.4 General Synthesis

#### 4.4.1 General Synthesis of Enamine Precursors

Starting aniline (0.5 mmol, ∼100 mg) was added to 1 mL EtOH, and the mixture was cooled to 0°C over an ice bath, under argon atmosphere, after which DEAD (1.1 eq, ∼103 µL) was added by needle through a septum. The reaction mixture was stirred at 0°C for 20 min and brought up to 50°C over 10 mins, held at 50°C and stirred for 3 h. The reaction crude was then dried by using a rotary evaporator at 50°C, with high vacuum. The dried mixture was then extracted in CH_2_Cl_2_ from 1.0 M HCl (aq) with three organic washes (5 mL) and dried again by using a rotary evaporator to obtain a faintly yellow gum.

#### 4.4.2 General Synthesis of Quinolones

Eaton's reagent (3 mL) was added directly to enamine precursor (0.5 mmol) at rt, and the flask was flushed extensively with argon and sealed under argon atmosphere. The reaction was then heated to 70°C and stirred for 2 h. The finished reaction crude was added dropwise to a stirring solution of 1.0 M NaCO_3_ (aq). Precipitated product was then vacuum filtered and washed with H_2_O, followed by minimal ice‐cold acetone. The retained solid was then dissolved in 10% DMSO/MeOH (v/v) and injected onto the Waters LC Prep AutoPurification System for final purification and separation of product isomers, where applicable.

#### 4.4.3 Hydrolysis of Quinolone Carboxylates

Quinolone ethyl carboxylates (**10**–**14**) were dissolved in 10% TEA/H_2_O (v/v) with MeCN added dropwise to solubilise and heated to 50°C for 2 h with stirring, dried in vacuo, redissolved in MeOH and injected onto a Waters LC Prep AutoPurification System for high purity (≥95%) isolation.

##### 4.4.3.1 7‐Bromo‐4‐Oxo‐1,4‐Dihydroquinoline‐2‐Carboxylic Acid (Caelestine A; 1)

White powder (18.2 mg, 14%). ^1^H NMR (800 MHz, DMF‐D_7_) δ_H_ 11.96 (1H, br s, N‐H), 8.33 (1H, d, *J* = 1.8 Hz, H‐8), 8.11 (1H, d, *J* = 8.6 Hz, H‐5), 7.57 (1H, dd, *J* = 8.6, 1.8 Hz, H‐6), 6.76 (1H, s, H‐3). ^13^C NMR (200 MHz, DMF‐D_7_) δ_C_ 178.6 (C‐4), 164.7 (2‐COOH), 142.6 (C‐8a), 141.2 (C‐2), 128.3 (C‐5), 128.0 (C‐6), 127.2 (C‐7), 126.1 (C‐4a), 123.1 (C‐8), 111.8 (C‐3). LCMS (ESI‐SQ, 25 eV) *m/z* (%): 270/268 (100/90)[*M* + H]^+^, 268/266 (100/95)[*M –* H]^−^, 224/222 (30/30)[*M* – COOH]^−^. HRMS (ESI‐qTOF) *m*/*z* calcd for C_10_H_7_^79^BrNO_3_^+^: 267.9604 [*M* + H]^+^; found: 267.9602, calcd for C_10_H_6_^79^BrNO_3_+Na^+^: 289.9423 [*M *+ Na]^+^; found 289.9421.

##### 4.4.3.2 6,7‐Dibromo‐4‐Oxo‐1,4‐Dihydroquinoline‐2‐Carboxylic Acid (Caelestine B; 2)

White powder (19.9 mg, 12%). ^1^H NMR (800 MHz, DMF‐D_7_) δ_H_ 12.09 (1H, br s, N‐H), 8.52 (1H, s, H‐8), 8.39 (1H, s, H‐5), 6.78 (1H, s, H‐3). ^13^C NMR (200 MHz, DMF‐D_7_) δ_C_ 177.6 (C‐4), 164.5 (2‐COOH), 142.0 (C‐2), 141.2 (C‐8a), 130.7 (C‐5), 129.2 (C‐7), 127.5 (C‐4a), 125.9 (C‐8), 119.9 (C‐6), 111.7 (C‐3). LCMS (ESI‐SQ, 25 eV) *m/z* (%): 350/348/346 (50/100/45)[*M* + H]^+^, 348/346/344 (50/100/25)[*M* – H]^−^, 304 (15)[*M* – COOH]^−^. HRMS (ESI‐qTOF) *m*/*z* calcd for C_10_H_5_^79^Br_2_NO_3_+Na^+^: 367.8528 [*M* + Na]^+^; found: 367.8524.

##### 4.4.3.3 6‐Bromo‐8‐Methoxy‐4‐Oxo‐1,4‐Dihydroquinoline‐2‐Carboxylic Acid (Caelestine C; 3)

White powder (34.3 mg, 23%). ^1^H NMR (800 MHz, DMF‐D_7_) δ_H_ 7.83 (1H, d, *J* = 2.0 Hz, H‐5), 7.54 (1H, d, *J* = 2.0 Hz, H‐7), 6.79 (1H, s, H‐3), 4.16 (3H, s, 8‐OCH_3_). ^13^C NMR (200 MHz, DMF‐D_7_) δ_C_ 177.3 (C‐4), 164.7 (2‐COOH), 151.3 (C‐8), 140.2 (C‐2), 131.2 (C‐8a), 128.4 (C‐4a), 119.6 (C‐5), 118.1 (C‐6), 116.1 (C‐7), 111.5 (C‐3), 58.0 (8‐OCH_3_). LCMS (ESI‐SQ, 25 eV) *m/z* (%): 300/298 (100/95)[*M* + H]^+^, 298/296 (60/100)[*M* – H]^−^, 254/252 (30/60)[*M* – COOH]^−^. HRMS (ESI‐qTOF) *m*/*z* calcd for C_11_H_9_^79^BrNO_4_^+^: 297.9709 [*M* + H]^+^; found 297.9706, calcd for C_11_H_8_^79^BrNO_4_+Na^+^: 319.9529 [*M* + Na]^+^; found: 319.9525.

##### 4.4.3.4 Diethyl 2‐((3‐Bromophenyl)amino)fumarate (7)

Yellow gum (167.7 mg, 98%). ^1^H NMR (800 MHz, DMSO‐D_6_) δ_H_ 9.56 (1H, s, N‐H), 7.24 (1H, m, H‐5), 7.23 (1H, m, H‐6), 7.18 (1H, m, H‐3), 6.92 (1H, m, H‐7), 5.37 (1H, s, H‐9), 4.15 (2H, q, *J* = 7.1 Hz, H‐16), 4.13 (2H, q, *J* = 7.1 Hz, H‐12), 1.21 (3H, t, *J* = 7.1 Hz, H‐13), 1.08 (3H, t, *J* = 7.1 Hz, H‐17). ^13^C NMR (200 MHz, DMSO‐D_6_) δ_C_ 167.4 (C‐10), 163.7 (C‐14), 146.2 (C‐8), 142.2 (C‐2), 130.8 (C‐6), 126.0 (C‐5), 122.7 (C‐3), 121.6 (C‐4), 119.1 (C‐7), 95.9 (C‐9), 62.1 (C‐16), 59.7 (C‐12), 14.2 (C‐13), 13.5 (C‐17). HRMS (ESI‐qTOF) *m*/*z* calcd for C_14_H_17_^79^BrNO_4_^+^: 342.0335 [*M* + H]^+^; found 342.0323, calcd for C_14_H_16_^79^BrNO_4_+Na^+^: 364.0155 [*M* + Na]^+^; found: 364.0141.

##### 4.4.3.5 Diethyl 2‐((3,4‐Dibromophenyl)amino)fumarate (8)

Yellow gum (193.7 mg, 92%). ^1^H NMR (800 MHz, DMSO‐D_6_) δ_H_ 9.53 (1H, s, N‐H), 7.62 (1H, d, *J* = 8.6 Hz, H‐6), 7.36 (1H, d, *J* = 2.7 Hz, H‐3), 6.87 (1H, dd, *J* = 8.6, 2.7 Hz, H‐7), 5.45 (1H, s, H‐9), 4.17 (2H, q, *J* = 7.1 Hz, H‐16), 4.13 (2H, q, *J* = 7.1 Hz, H‐12), 1.21 (3H, t, *J* = 7.1 Hz, H‐13), 1.11 (3H, t, *J* = 7.1 Hz, H‐17). ^13^C NMR (200 MHz, DMSO‐D_6_) δ_C_ 167.1 (C‐10), 163.6 (C‐14), 145.3 (C‐8), 141.4 (C‐2), 133.6 (C‐6), 124.6 (C‐3), 123.8 (C‐4), 120.8 (C‐7), 117.1 (C‐5), 97.1 (C‐9), 62.2 (C‐16), 59.8 (C‐12), 14.2 (C‐13), 13.6 (C‐17). HRMS (ESI‐qTOF) *m*/*z* calcd for C_14_H_16_^79^Br_2_NO_4_^+^: 419.9441 [*M* + H]^+^; found 419.9424, calcd for C_14_H_15_^79^Br_2_NO_4_+Na^+^: 441.9260 [*M* + Na]^+^; found: 441.9241.

##### 4.4.3.6 Diethyl 2‐((4‐Bromo‐2‐Methoxyphenyl)amino)fumarate (9)

Yellow gum (173.1 mg, 93%). ^1^H NMR (800 MHz, DMSO‐D_6_) δ_H_ 9.54 (1H, s, N‐H), 7.21 (1H, d, *J* = 2.1 Hz, H‐4), 7.07 (1H, dd, *J* = 8.5, 2.1 Hz, H‐6), 6.78 (1H, d, *J* = 8.5 Hz, H‐7), 5.28 (1H, s, H‐9), 4.15 (2H, q, *J* = 7.1 Hz, H‐16), 4.13 (2H, q, *J* = 7.1 Hz, H‐12), 3.81 (3H, s, H‐19), 1.22 (3H, t, *J* = 7.1 Hz, H‐13), 1.12 (3H, t, *J* = 7.1 Hz, H‐17). ^13^C NMR (200 MHz, DMSO‐D_6_) δ_C_ 168.5 (C‐10), 163.1 (C‐14), 151.0 (C‐3), 147.5 (C‐8), 128.1 (C‐2), 123.2 (C‐6), 121.7 (C‐7), 116.0 (C‐5), 114.6 (C‐4), 92.3 (C‐9), 61.9 (C‐16), 59.7 (C‐12), 56.1 (C‐19), 14.2 (C‐13), 13.6 (C‐17). HRMS (ESI‐qTOF) *m*/*z* calcd for C_15_H_19_^79^BrNO_5_^+^: 372.0441 [*M* + H]^+^; found 372.0427, calcd for C_15_H_18_^79^BrNO_5_+Na^+^: 394.0261 [*M* + Na]^+^; found: 394.0245.

##### 4.4.3.7 Ethyl 7‐Bromo‐4‐Oxo‐1,4‐Dihydroquinoline‐2‐Carboxylate (10)

White powder (20.3 mg, 14%). ^1^H NMR (800 MHz, DMF‐D_7_) δ_H_ 12.08 (1H, br s, N‐H), 8.28 (1H, d, *J* = 1.8 Hz, H‐8), 8.10 (1H, d, *J* = 8.6 Hz, H‐5), 7.58 (1H, dd, *J* = 8.6, 1.8 Hz, H‐6), 6.71 (1H, s, H‐3), 4.46 (2H, q, *J* = 7.1 Hz, H‐11), 1.40 (3H, t, *J* = 7.1 Hz, H‐12). ^13^C NMR (200 MHz, DMF‐D_7_) δ_C_ 178.6 (C‐4), 163.3 (C‐9), 142.4 (C‐8a), 139.7 (C‐2), 128.3 (C‐5), 128.2 (C‐6), 127.5 (C‐7), 126.3 (C‐4a), 122.9 (C‐8), 112.1 (C‐3), 63.9 (C‐11), 14.6 (C‐12). LCMS (ESI‐SQ, 25 eV) *m/z* (%): 298/296 (100/95)[*M* + H]^+^, 296/294 (100/80)[*M* – H]^−^. HRMS (ESI‐qTOF) *m*/*z* calcd for C_12_H_11_^79^BrNO_3_: 295.9917 [*M* + H]^+^; found 295.9923.

##### 4.4.3.8 Ethyl 5‐Bromo‐4‐Oxo‐1,4‐Dihydroquinoline‐2‐Carboxylate (11)

White powder (39.2 mg, 27%). ^1^H NMR (800 MHz, DMF‐D_7_) δ_H_ 12.03 (1H, s, N‐H), 8.05 (1H, dd, *J* = 8.3, 0.8 Hz, H‐8), 7.61 (1H, dd, *J* = 7.3, 0.8 Hz, H‐6), 7.57 (1H, dd, *J* = 8.3, 7.3 Hz, H‐7), 6.68 (1H, s, H‐3), 4.45 (2H, q, *J* = 7.1 Hz, H‐11), 1.39 (3H, t, *J* = 7.1 Hz, H‐12). ^13^C NMR (200 MHz, DMF‐D_7_) δ_C_ 178.2 (C‐4), 163.3 (C‐9), 144.1 (C‐8a), 138.2 (C‐2), 133.6 (C‐7), 131.6 (C‐6), 124.0 (C‐4a), 120.7 (C‐5), 120.6 (C‐8), 113.2 (C‐3), 63.8 (C‐11), 14.6 (C‐12). LCMS (ESI‐SQ, 25 eV) *m/z* (%): 298/296 (100/95)[*M* + H]^+^, 296/294 (100/80)[*M* – H]^−^. HRMS (ESI‐qTOF) *m*/*z* calcd for C_12_H_11_^79^BrNO_3_: 295.9917 [*M* + H]^+^; found 295.9923, calcd for C_12_H_10_^79^BrNO_3_+Na^+^: 317.9736 [*M* + Na]^+^; found: 317.9742.

##### 4.4.3.9 Ethyl 6,7‐Dibromo‐4‐Oxo‐1,4‐Dihydroquinoline‐2‐Carboxylate (12)

White powder (22.4 mg, 13%). ^1^H NMR (800 MHz, DMF‐D_7_) δ_H_ 12.21 (1H, br s, N‐H), 8.46 (1H, s, H‐8), 8.38 (1H, s, H‐5), 6.75 (1H, s, H‐3), 4.47 (2H, q, *J* = 7.2 Hz, H‐11), 1.40 (3H, t, *J* = 7.2 Hz, H‐12). ^13^C NMR (200 MHz, DMF‐D_7_) δ_C_ 177.5 (C‐4), 163.3 (C‐9), 141.2 (C‐8a), 140.1 (C‐2), 130.7 (C‐5), 129.5 (C‐7), 127.6 (C‐4a), 125.8 (C‐8), 120.3 (C‐6), 112.1 (C‐3), 64.0 (C‐11), 14.6 (C‐12). LCMS (ESI‐SQ, 25 eV) *m/z* (%): 378/376/374 (55/100/45) [*M* + H]^+^, 376/374/372 (45/100/50)[*M* – H]^−^. HRMS (ESI‐qTOF) *m*/*z* calcd for C_12_H_10_^79^Br_2_NO_3_^+^: 373.9022 [*M* + H]^+^; found 373.9028.

##### 4.4.3.10 Ethyl 5,6‐Dibromo‐4‐Oxo‐1,4‐Dihydroquinoline‐2‐Carboxylate (13)

White powder (44.9 mg, 26%). ^1^H NMR (800 MHz, DMF‐D_7_) δ_H_ 12.16 (1H, br s, N‐H), 8.04 (1H, d,*J* = 9.0 Hz, H‐7), 8.02 (1H, d, *J* = 9.0 Hz, H‐8), 6.74 (1H, s, H‐3), 4.45 (2H, q, *J* = 7.2 Hz, H‐11), 1.39 (3H, t, *J* = 7.2 Hz, H‐12). ^13^C NMR (200 MHz, DMF‐D_7_) δ_C_ 177.3 (C‐4), 163.3 (C‐9), 143.0 (C‐8a), 138.2 (C‐2), 137.4 (C‐7), 126.1 (C‐4a), 124.3 (C‐6), 122.7 (C‐5), 122.1 (C‐8), 113.5 (C‐3), 63.9 (C‐11), 14.6 (C‐12). LCMS (ESI‐SQ, 25 eV) *m/z* (%): 378/376/374 (45/100/50)[*M* + H]^+^, 376/374/372 (55/100/45)[*M* – H]^−^. HRMS (ESI‐qTOF) *m*/*z* calcd for C_12_H_10_^79^Br_2_NO_3_^+^: 373.9022 [*M* + H]^+^; found 373.9028.

##### 4.4.3.11 Ethyl 6‐Bromo‐8‐Methoxy‐4‐Oxo‐1,4‐Dihydroquinoline‐2‐Carboxylate (14)

White powder (37.9 mg, 25%). ^1^H NMR (800 MHz, DMF‐D_7_) δ_H_ 9.88 (1H, br s, N‐H), 7.80 (1H, s, H‐7), 7.50 (1H, s, H‐5), 6.70 (1H, s, H‐3), 4.48 (2H, q, *J* = 7.1 Hz, H‐11), 4.13 (3H, s, H‐14), 1.42 (3H, t, *J* = 7.1 Hz, H‐12). ^13^C NMR (200 MHz, DMF‐D_7_) δ_C_ 177.5 (C‐4), 163.3 (C‐9), 151.1 (C‐8), 138.6 (C‐2), 130.8 (C‐8a), 128.5 (C‐4a), 119.5 (C‐5), 118.3 (C‐6), 116.3 (C‐7), 112.0 (C‐3), 64.2 (C‐11), 57.9 (C‐14), 14.6 (C‐12). LCMS (ESI‐SQ, 25 eV) *m/z* (%): 328/326 (100/90)[*M* + H]^+^, 326/324 (95/100)[*M* – H]^−^. HRMS (ESI‐qTOF) *m*/*z* calcd for C_13_H_13_^79^BrNO_4_^+^: 326.0022 [*M* + H]^+^; found 326.0027.

##### 4.4.3.12 5‐Bromo‐4‐Oxo‐1,4‐Dihydroquinoline‐2‐Carboxylic Acid (15)

White powder (34.8 mg, 25%). ^1^H NMR (800 MHz, DMF‐D_7_) δ_H_ 11.89 (1H, s, N‐H), 8.10 (1H, dd, *J* = 8.3, 1.1 Hz, H‐8), 7.60 (1H, dd, *J* = 7.6, 1.1 Hz, H‐6), 7.56 (1H, dd, *J* = 8.3, 7.6 Hz, H‐7), 6.71 (1H, s, H‐3). ^13^C NMR (200 MHz, DMF‐D_7_) δ_C_ 178.3 (C‐4), 164.6 (2‐COOH), 144.2 (C‐8a), 139.4 (C‐2), 133.5 (C‐7), 131.5 (C‐6), 123.9 (C‐4a), 120.8 (C‐8), 120.7 (C‐5), 113.1 (C‐3). LCMS (ESI‐SQ, 25 eV) *m/z* (%): 270/268 (100/90)[*M* + H]^+^, 268/266 (30/70)[*M* – H]^−^, 224/222 (100/100)[*M*‐COOH]^−^. HRMS (ESI‐qTOF) *m*/*z* calcd for C_10_H_6_^79^BrNO_3_+Na^+^: 289.9423 [*M* + Na]^+^; found: 289.9418.

##### 4.4.3.13 5,6‐Dibromo‐4‐Oxo‐1,4‐Dihydroquinoline‐2‐Carboxylic Acid (16)

White powder (39.8 mg, 23%). ^1^H NMR (800 MHz, DMF‐D_7_) δ_H_ 8.09 (1H, d, *J* = 9.0 Hz, H‐8), 7.99 (1H, d, *J* = 9.0 Hz, H‐7), 6.74 (1H, s, H‐3). ^13^C NMR (200 MHz, DMF‐D_7_) δ_C_ 178.2 (C‐4), 164.4 (2‐COOH), 146.9 (C‐2), 142.5 (C‐8a), 136.2 (C‐7), 125.7 (C‐4a), 122.8 (C‐6), 122.7 (C‐5), 122.1 (C‐8), 111.2 (C‐3). LCMS (ESI‐SQ, 25 eV) *m/z* (%): 350/348/346 (50/100/50)[*M* + H]^+^, 348/346/344 (45/85/50)[*M* – H]^−^, 304/302/300 (60/100/30)[*M* – COOH]^−^. HRMS (ESI‐qTOF) *m*/*z* calcd for C_10_H_4_^79^Br_2_NO_3_+Na^+^: 367.8528 [*M* + Na]^+^; found: 367.8525.

## Funding

This study was supported by the Australian Research Council (LE0668477, LE140100119, LE0237908, and LE230100128).

## Conflicts of Interest

The authors declare no conflicts of interest.

## Supporting information

Supplementary Material

## Data Availability

The data that support the findings of this study are available from the corresponding author upon reasonable request.
