# Supplementary material for: Total Synthesis and Antimalarial Studies of Caelestines A–C
Source: ChemMedChem. 2026 Jun 15;21(11):e70345. doi: 10.1002/cmdc.70345 (PMC13268815; doi:10.1002/cmdc.70345)
Supplement: Supplementary file 1 — Supplementary Material [file CMDC-21-e70345-s001.pdf]

Total Synthesis and Antimalarial Studies of Caelestines A–C

Supporting Information

Henry S.T. Smith, Aaron Lock, Vicky M. Avery, and Rohan A. Davis*

H. S.T. Smith, R. A. Davis

Institute for Biomedicine and Glycomics
Griffith University
46 Don Young Road, Nathan, Queensland, 4111, Australia
E-mail: r.davis@griffith.edu.au

A. Lock, V. M. Avery

Discovery Biology
Griffith University
46 Don Young Road, Nathan, Queensland, 4111, Australia

R. A. Davis

NatureBank
Griffith University
46 Don Young Road, Nathan, Queensland, 4111, Australia

H. S.T. Smith, A. Lock, V. M. Avery, R. Davis

School of Environment and Science
Griffith University
170 Kessels Road, Nathan QLD 4111, Australia

Table of Contents

S1: NMR Data Table for Caelestine A (**1**)[a] 1

S2: ^{1}H NMR Spectrum of Caelestine A (**1**) in DMF-D_7 2

S3: ^{1}H NMR Comparison of Synthetic Caelestine A (**1**) with Authentic Natural Product Sample in MeOH-D_4 3

S4: ^{13}C NMR spectrum of Caelestine A (**1**) in DMF-D_7 4

S5: NMR Data Table for Caelestine B (**2**)[a] 5

S6: ^{1}H NMR Spectrum of Caelestine B (**2**) in DMF-D_7 6

S7: ^{1}H NMR Comparison of Synthetic Caelestine B (**2**) with Authentic Natural Product Sample in MeOH-D_4 7

S8: ^{13}C NMR spectrum of Caelestine B (**2**) in DMF-D_7 8

S9: NMR Data Table for Caelestine C (**3**)[a] 9

S10: ^{1}H NMR Spectrum of Caelestine C (**3**) in DMF-D_7 10

S11: ^{1}H NMR Comparison of Synthetic Caelestine C (**3**) with Authentic Natural Product Sample in MeOH-D_4 11

S12: ^{13}C NMR spectrum of Caelestine C (**3**) in DMF-D_7 12

S13: NMR Data Table for Compound **7**[a] 13

S14: ^{1}H NMR Spectrum of Compound **7** in DMSO-D_6 14

S15: ^{13}C NMR Spectrum of Compound **7** in DMSO-D_6 15

S16: NMR Data Table for Compound **8**[a] 16

S17: ^{1}H NMR Spectrum of Compound **8** in DMSO-D_6 17

S18: ^{13}C NMR Spectrum of Compound **8** in DMSO-D_6 18

S19: NMR Data Table for Compound **9**[a] 19

S20: ^{1}H NMR Spectrum of Compound **9** in DMSO-D_6 20

S21: ^{13}C NMR Spectrum of Compound **9** in DMSO-D_6 21

S22: NMR Data Table for Compound **10**[a] 22

S23: ^{1}H NMR Spectrum of Compound **10** in DMF-D_7 23

S24: ^{13}C NMR Spectrum of Compound **10** in DMF-D_7 24

S25: NMR Data Table for Compound **11**[a] 25

S26: ^{1}H NMR Spectrum of Compound **11** in DMF-D_7 26

S27: ^{13}C NMR Spectrum of Compound **11** in DMF-D_7 27

S28: NMR Data Table for Compound **12**[a] 28

S29: ^{1}H NMR Spectrum of Compound **12** in DMF-D_7 29
S30: ^{13}C NMR Spectrum of Compound **12** in DMF-D_7 30
S31: NMR Data Table for Compound **13**[a] 31
S32: ^{1}H NMR Spectrum of Compound **13** in DMF-D_7 32
S33: ^{13}C NMR Spectrum of Compound **13** in DMF-D_7 33
S34: NMR Data Table for Compound **14**[a] 34
S35: ^{1}H NMR Spectrum of Compound **14** in DMF-D_7 35
S36: ^{13}C NMR Spectrum of Compound **14** in DMF-D_7 36
S37: NMR Data Table for Compound **15**[a] 37
S38: ^{1}H NMR Spectrum of Compound **15** in DMF-D_7 38
S39: ^{13}C NMR Spectrum of Compound **15** in DMF-D_7 39
S40: NMR Data Table for Compound **16**[a] 40
S41: ^{1}H NMR Spectrum of Compound **16** in DMF-D_7 41
S42: ^{13}C NMR Spectrum of Compound **16** in DMF-D_7 42
S43: Biological Data 43
S44: Chromatography Studies 44

S1: NMR Data Table for Caelestine A (**1**)[a]

Position	δ_H, mult. (J in Hz), int.	δ_C, mult.	COSY	HMBC
1	11.96, s, 1H			
2		141.2, C		
3	6.76, s, 1H	111.8, CH		2[w], 4a, 2-COOH
4		178.6, C		
4a		126.1, C		
5	8.11, d (8.6), 1H	128.3, CH	6, 8[w]	4, 7, 8a
6	7.57, dd (8.6, 1.8), 1H	128.0, CH	5[w], 8	4a, 8
7		127.2, C		
8	8.33, d (1.8), 1H	123.1, CH	6	4a, 6, 8a[w]
8a		142.6 C		
2-COOH		164.7, C		

[a] Recorded in DMF-D_7, 800 MHz (^{1}H NMR) and 200 MHz (^{13}C NMR) at 25 °C; [w] Weak.

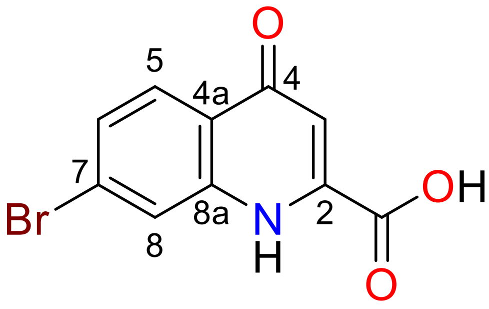

S2: ^{1}H NMR Spectrum of Caelestine A (**1**) in DMF-D$_7$

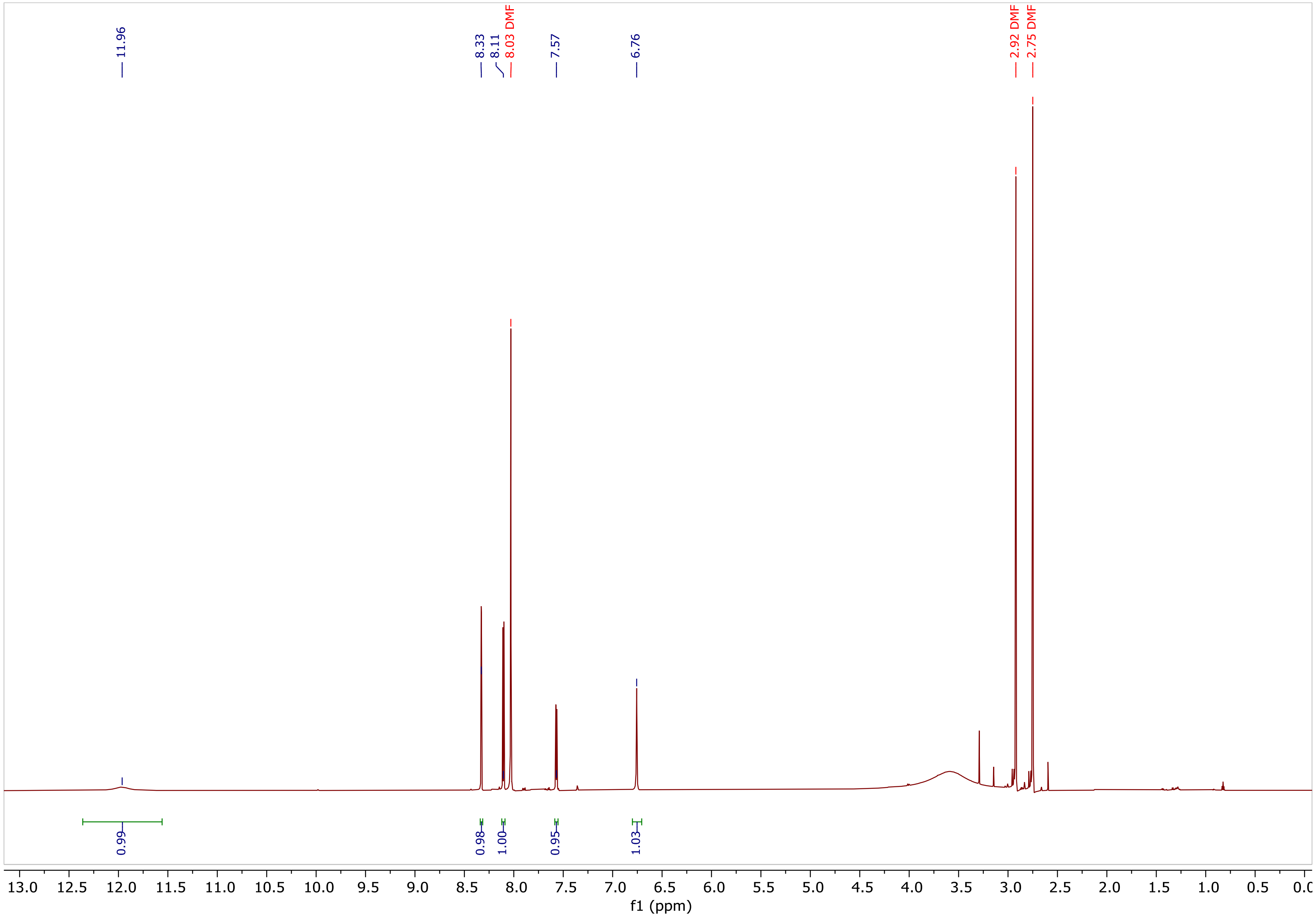

S3: ^{1}H NMR Comparison of Synthetic Caelestine A (**1**) with Authentic Natural Product Sample in MeOH-D4

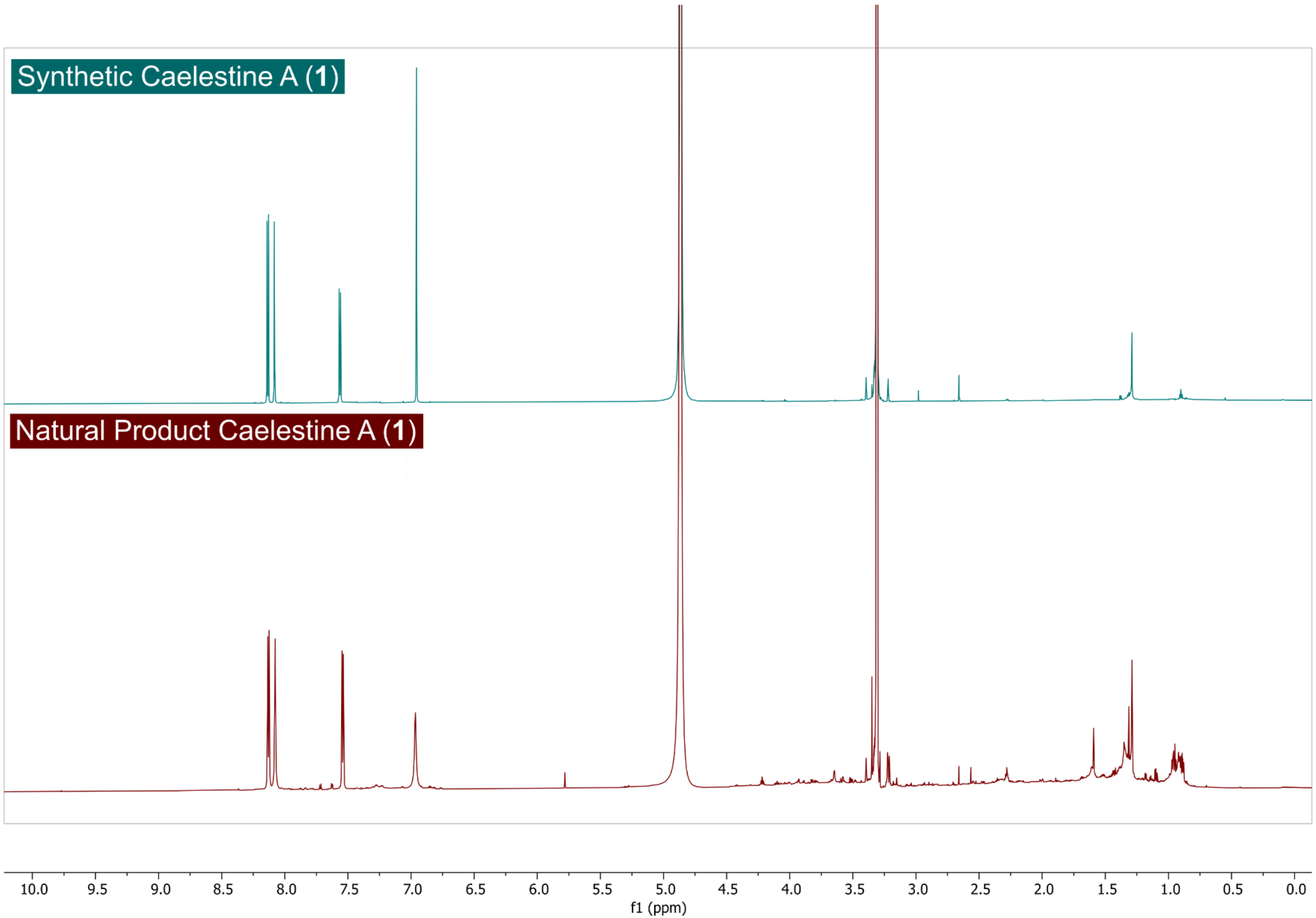

S4: ^{13}C NMR Spectrum of Caelestine A (**1**) in DMF-D_7

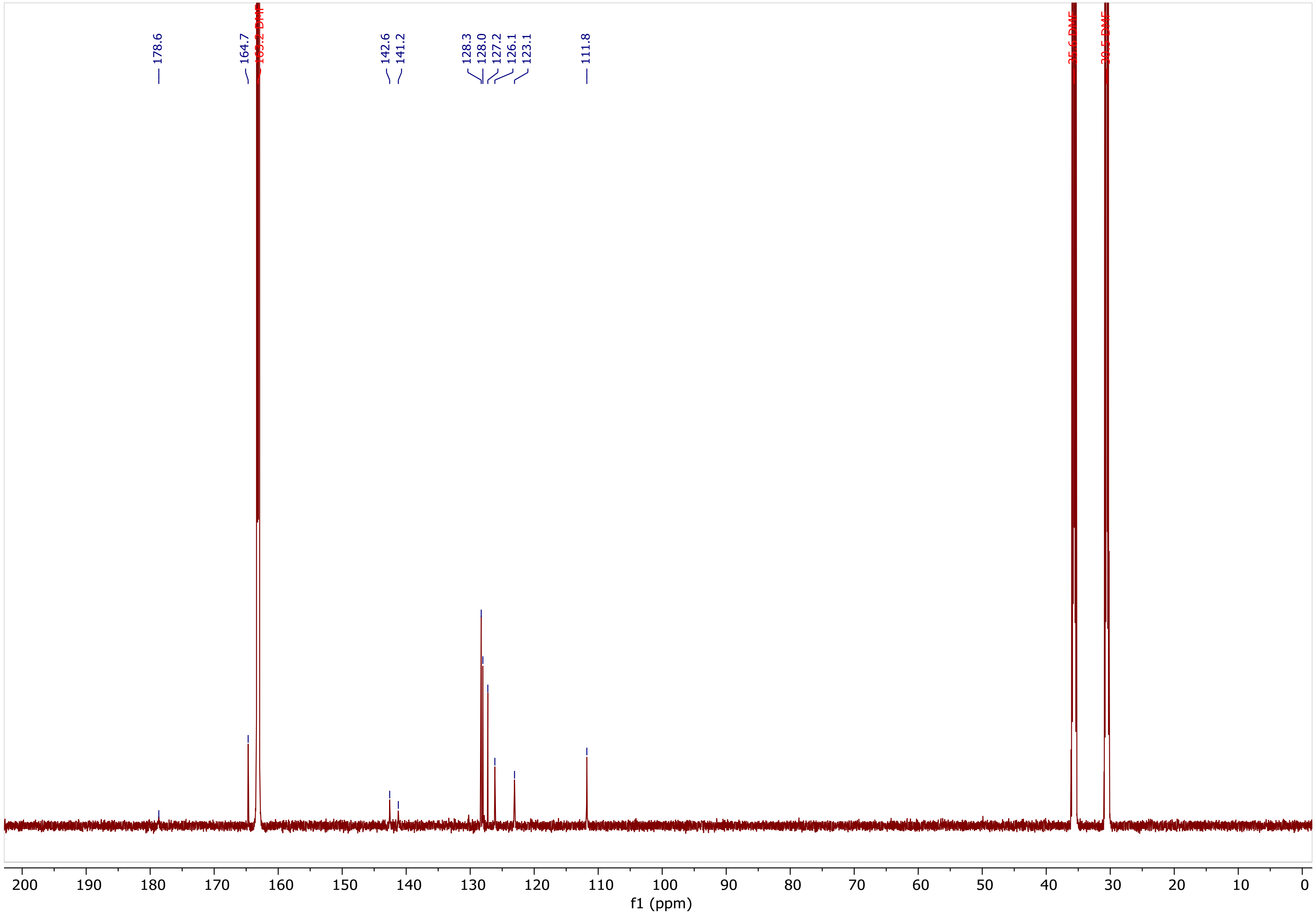

S5: NMR Data Table for Caelestine B (**2**)[a]

Position	δ_H, mult. (J in Hz), int.	δ_C, mult.	COSY	HMBC
1	12.09, s, 1H			
2		142.0, C		
3	6.78, s, 1H	111.7, CH		2[w], 4a, 2-COOH
4		177.6, C		
4a		127.5, C		
5	8.39, s, 1H	130.7, CH		4, 7, 8a
6		119.9, C		
7		129.2, C		
8	8.52, s, 1H	125.9, CH		4a, 6, 8a[w]
8a		141.2 C		
2-COOH		164.5, C		

[a] Recorded in DMF-D_7, 800 MHz (^{1}H NMR) and 200 MHz (^{13}C NMR) at 25 °C with 2% MeOH-D_4; [w] Weak.

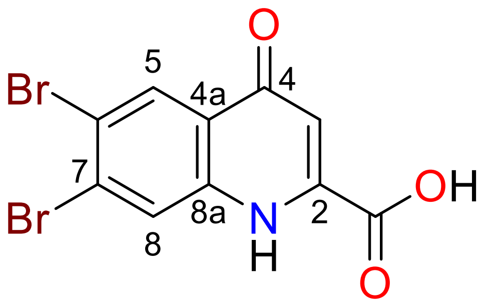

S6: ^{1}H NMR Spectrum of Caelestine B (**2**) in DMF-D$_7$

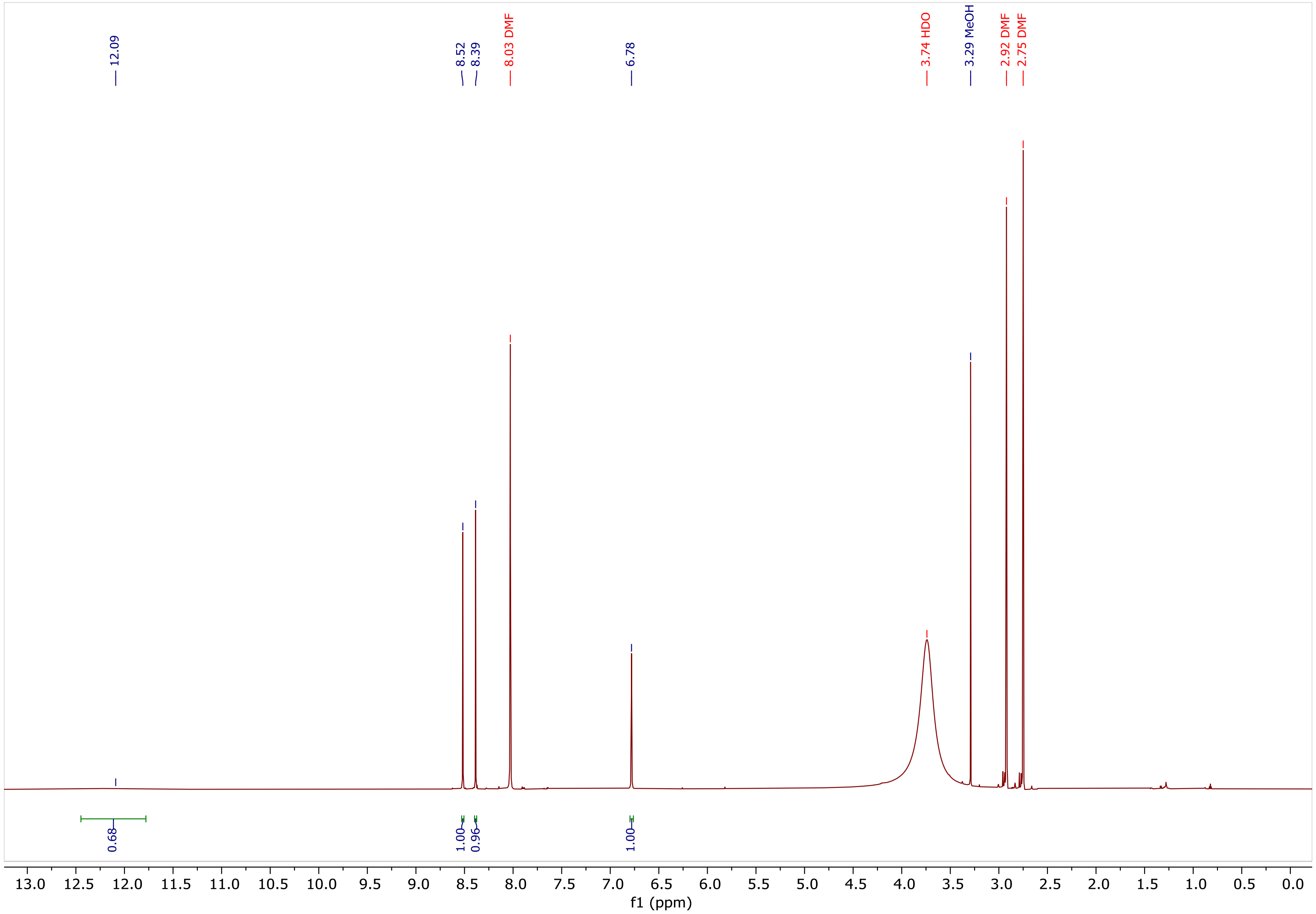

S7: ^{1}H NMR Comparison of Synthetic Caelestine B (**2**) with Authentic Natural Product Sample in MeOH-D$_4$

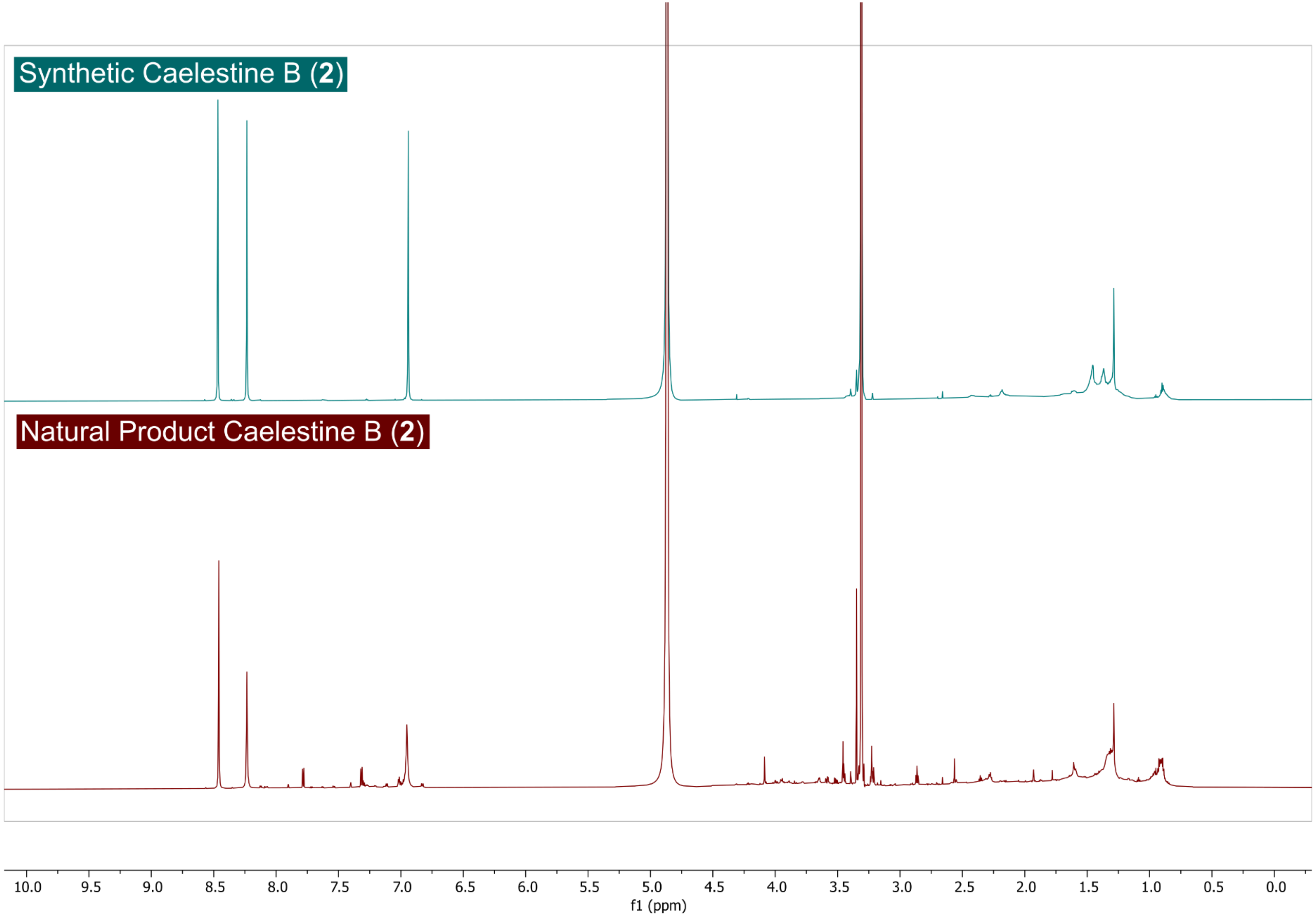

S8: ^{13}C NMR spectrum of Caelestine B (**2**) in DMF-D_7

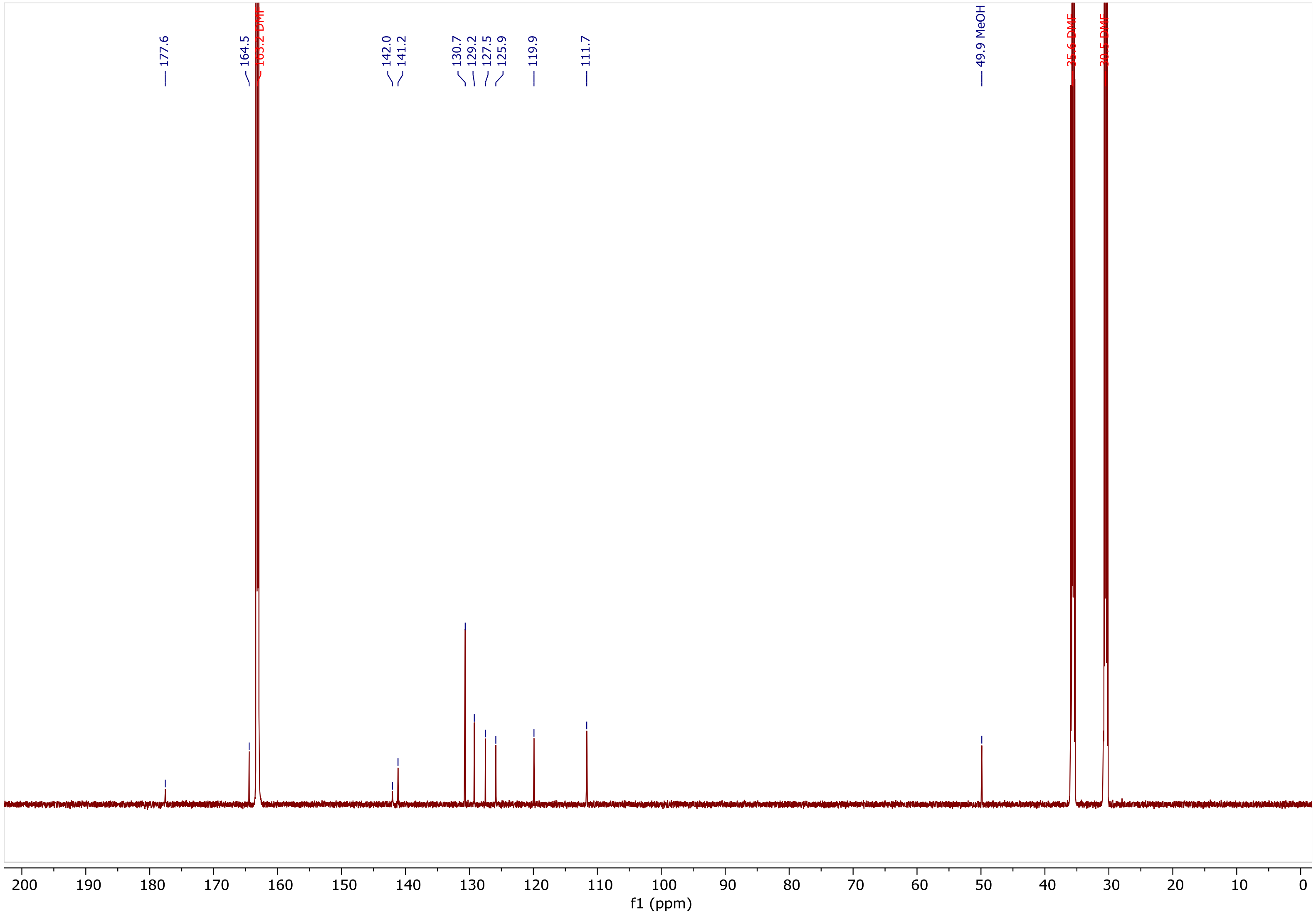

S9: NMR Data Table for Caelestine C (**3**)[a]

Position	δ_H, mult. (*J* in Hz), int.	δ_C, mult.	COSY	HMBC
1	ND			
2		140.2, C		
3	6.79, s, 1H	111.5, CH		4a, 2-COOH
4		177.3, C		
4a		128.4, C		
5	7.83, d (2.0), 1H	119.6, CH	7	4, 4a, 6, 7
6		118.1, C		
7	7.54, d (2.0), 1H	116.1, CH	5	5, 6, 8, 8a
8		151.3, C		
8a		131.2, C		
2-COOH		164.7, C		
8-OMe	4.16, s, 3H	58.0, CH_3		8

[a] Recorded in DMF-D_7, 800 MHz (^{1}H NMR) and 200 MHz (^{13}C NMR) at 25 °C; ND Not Detected; [w] Weak.

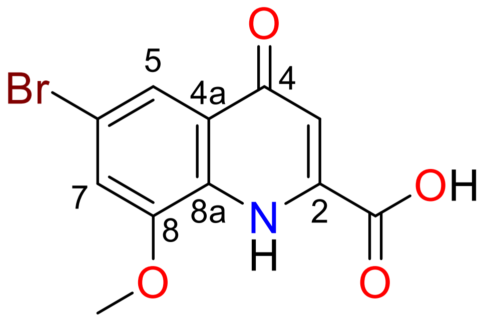

S10: ^{1}H NMR Spectrum of Caelestine C (**3**) in DMF-D$_7$

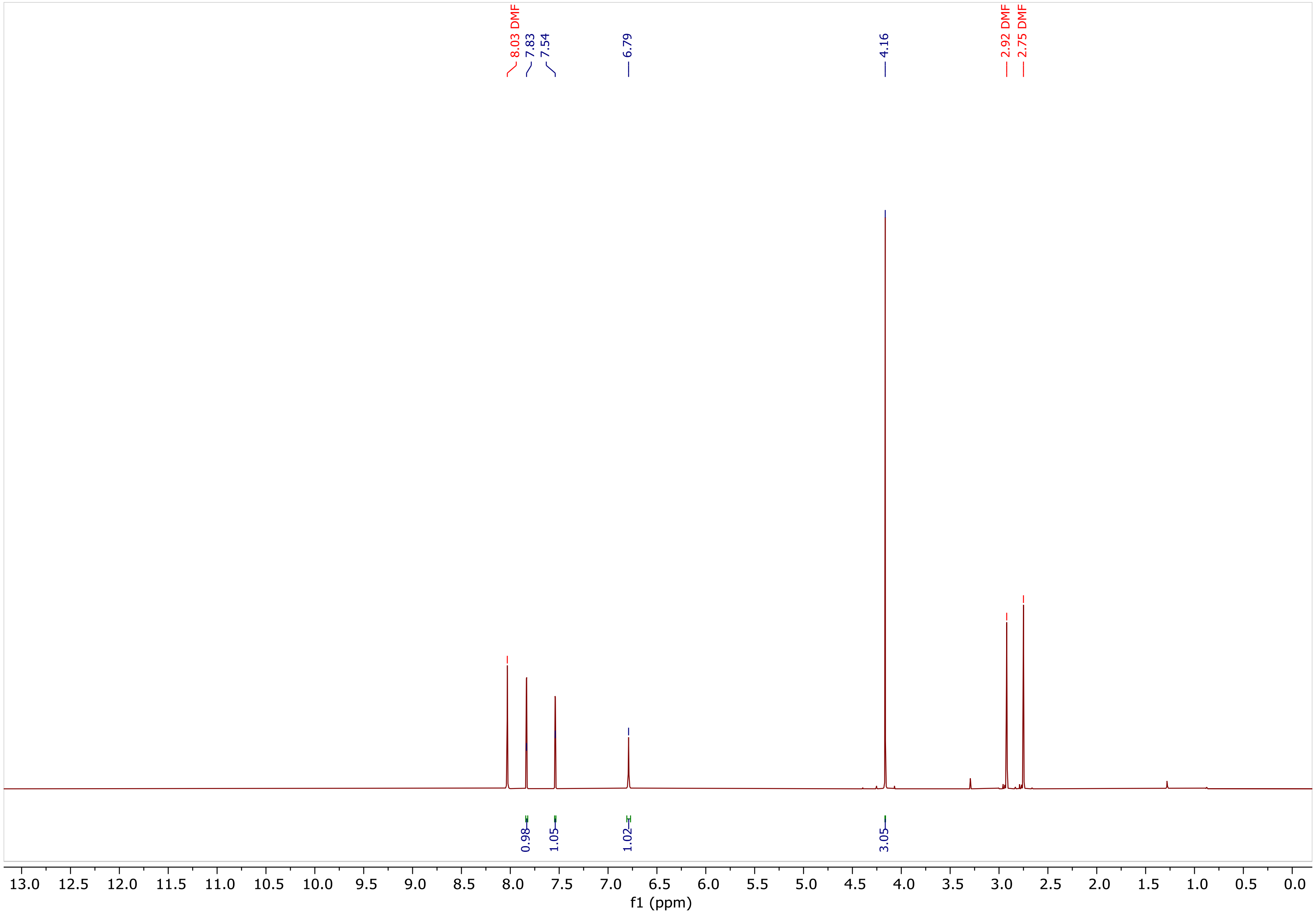

S11: ^{1}H NMR Comparison of Synthetic Caelestine C (**3**) with Authentic Natural Product Sample in MeOH-D_4

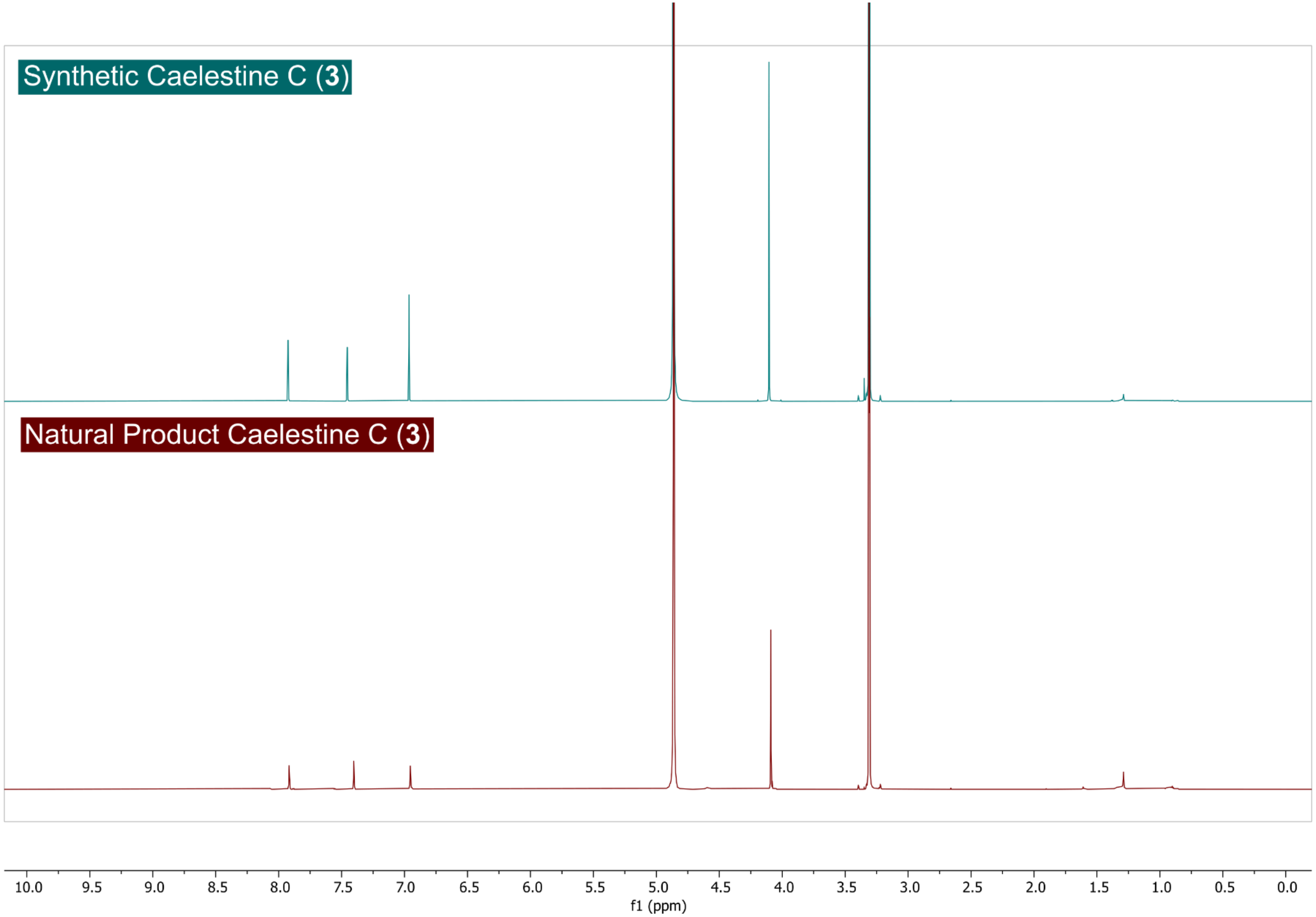

S12: ^{13}C NMR spectrum of Caelestine C (**3**) in DMF-D_7

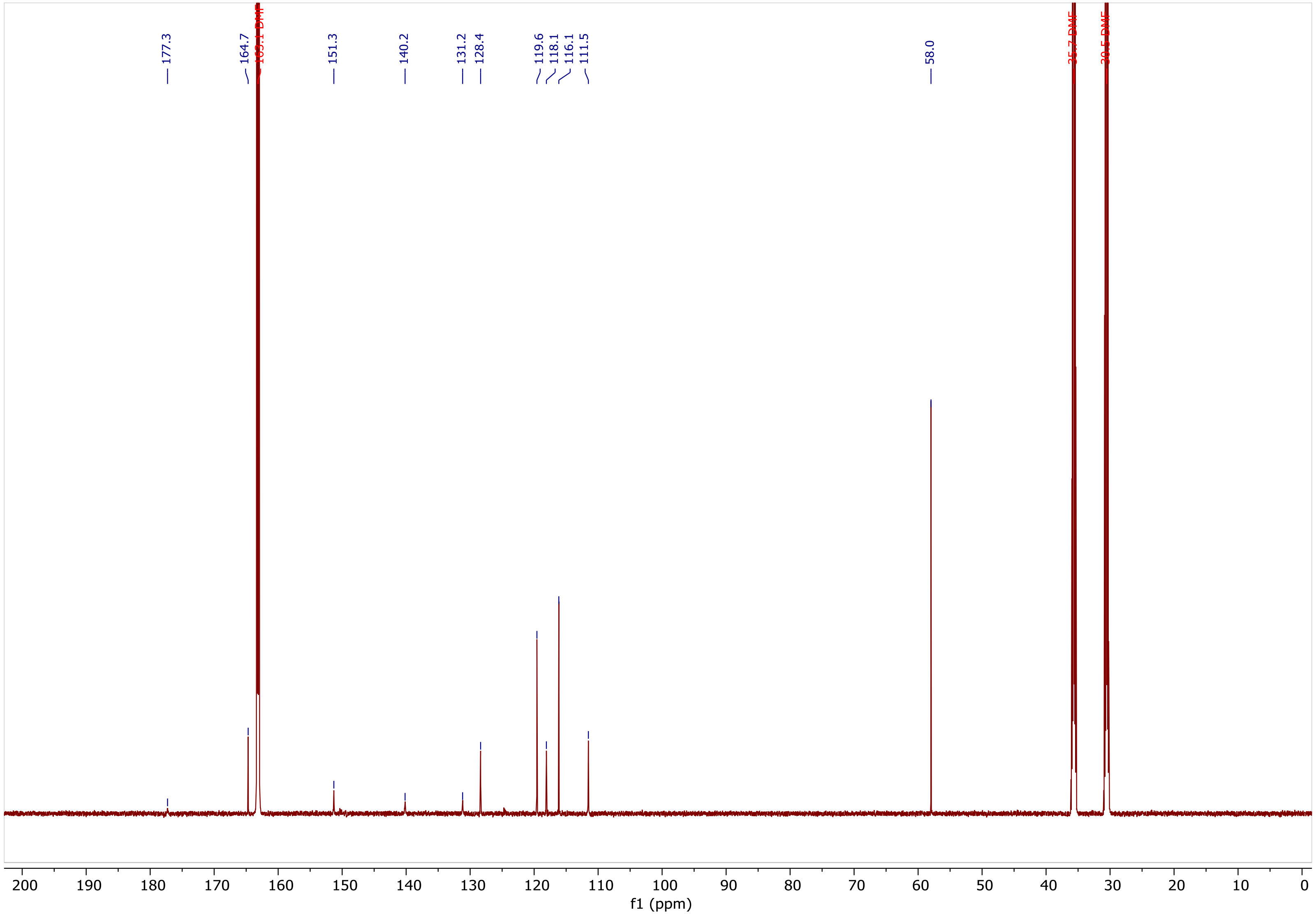

S13: NMR Data Table for Compound **7**[a]

Position	δ_H, mult. (*J* in Hz), int.	δ_C, mult.	COSY	HMBC
1	9.56, s, 1H			3, 7, 9, 14
2		142.2, C		
3	7.18, m, 1H	122.7, CH	5^w, 7^w	5, 7
4		121.6, C		
5	7.24, m, 1H	126.0, CH	6	3, 7
6	7.23, m, 1H	130.8, CH	5, 7	2, 4
7	6.92, m, 1H	119.1, CH	6	3, 5
8		146.2, C		
9	5.37, s, 1H	95.9, CH		14
10		167.4, C		
12	4.13, q (7.1), 2H	59.7, CH_2	13	10, 13
13	1.21, t (7.1), 3H	14.2, CH_3	12	12
14		163.7, C		
16	4.15, q (7.1), 2H	62.1, CH_2	17	14, 17
17	1.08, t (7.1), 3H	13.5, CH_3	16	16

[a] Recorded in DMSO-D_6, 800 MHz (^{1}H NMR) and 200 MHz (^{13}C NMR) at 25 °C; [w] Weak.

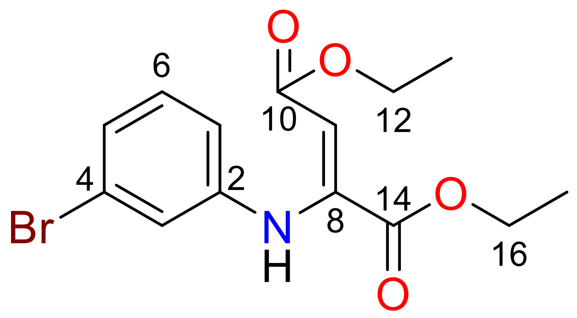

S14: ^{1}H NMR Spectrum of Compound **7** in DMSO-D$_6$

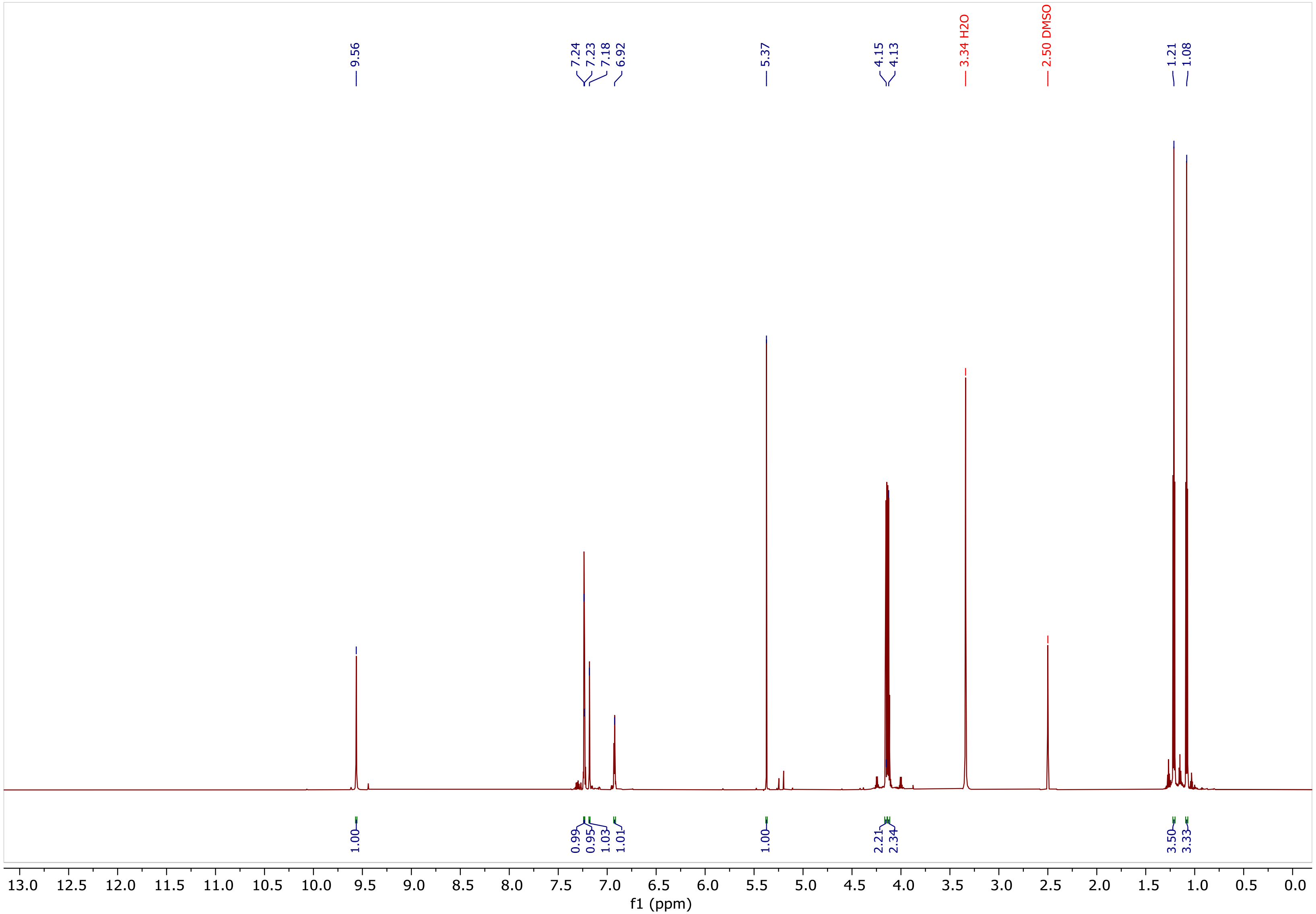

S15: ^{13}C NMR Spectrum of Compound **7** in DMSO-D$_6$

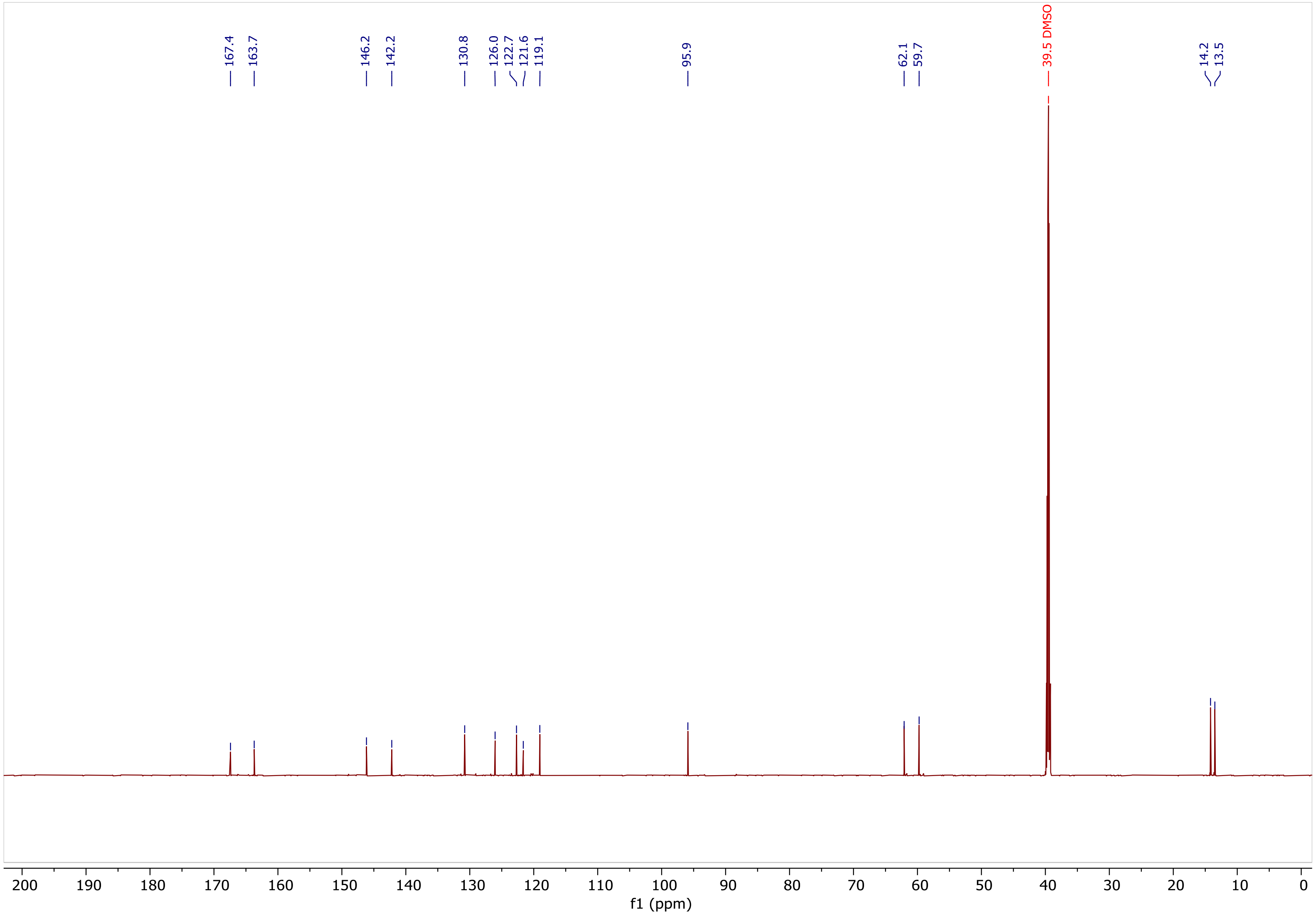

S16: NMR Data Table for Compound **8**[a]

Position	δ_H, mult. (*J* in Hz), int.	δ_C, mult.	COSY	HMBC
1	9.53, s, 1H			3, 7, 9, 14
2		141.4, C		
3	7.36, d (2.7), 1H	124.6, CH	7	5, 7
4		123.8, C		
5		117.1, C		
6	7.62, d (8.6), 1H	133.6, CH	7	2, 4
7	6.87, dd (8.6, 2.7), 1H	120.8, CH	3, 6	3, 5
8		145.3, C		
9	5.45, s, 1H	97.1, CH		14
10		167.1, C		
12	4.13, q (7.1), 2H	59.8, CH_2	13	10, 13
13	1.21, t (7.1), 3H	14.2, CH_3	12	12
14		163.6, C		
16	4.17, q (7.1), 2H	62.2, CH_2	17	14, 17
17	1.11, t (7.1), 3H	13.6, CH_3	16	16

[a] Recorded in DMSO-D_6, 800 MHz (^{1}H NMR) and 200 MHz (^{13}C NMR) at 25 °C; [w] Weak.

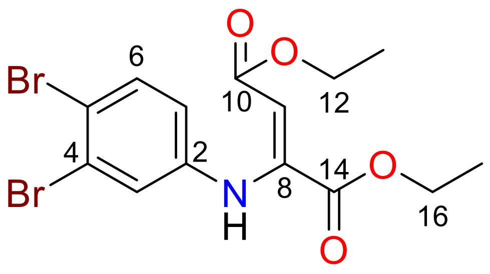

S17: ^{1}H NMR Spectrum of Compound **8** in DMSO-D$_6$

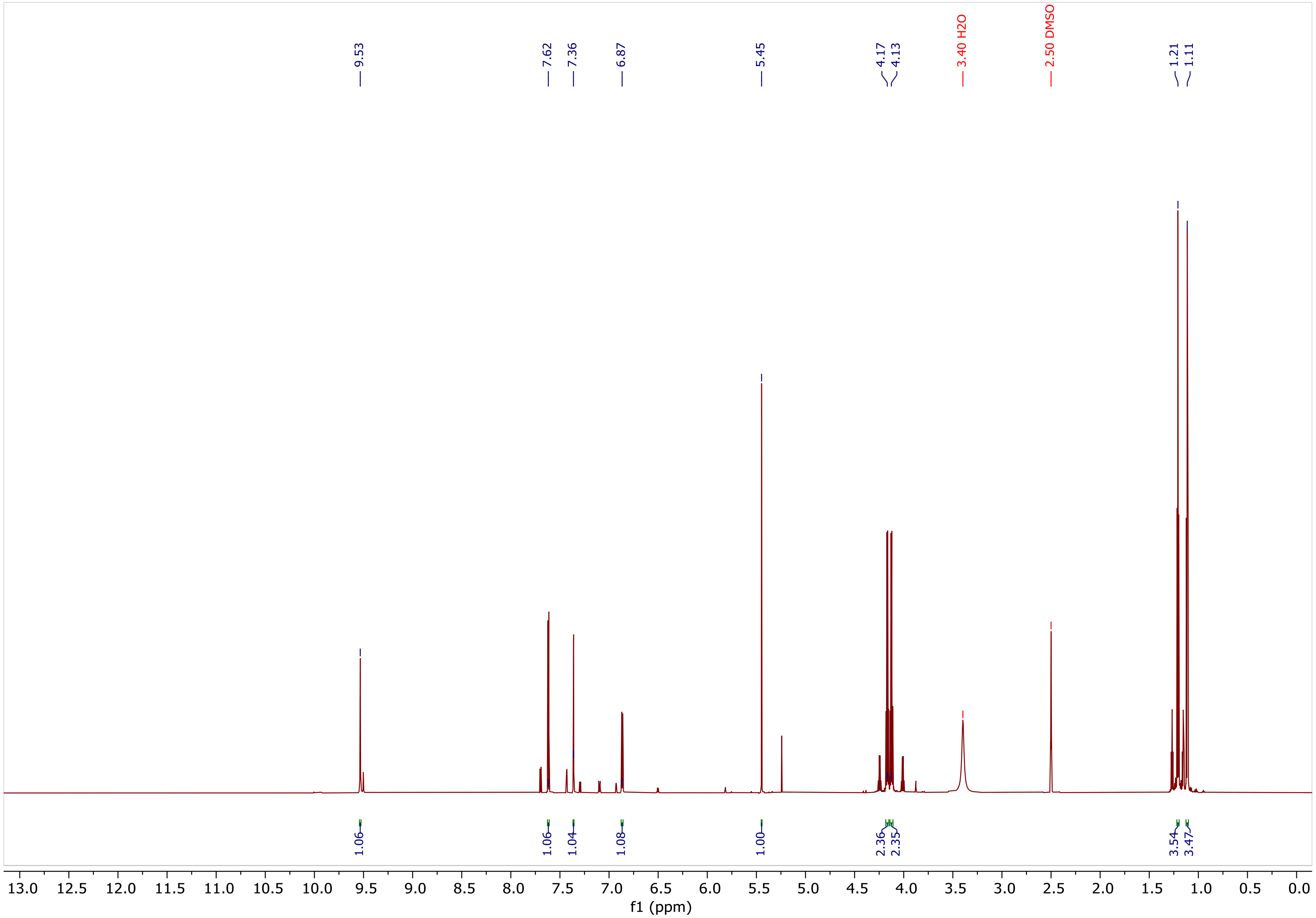

S18: ^{13}C NMR Spectrum of Compound **8** in DMSO-D$_6$

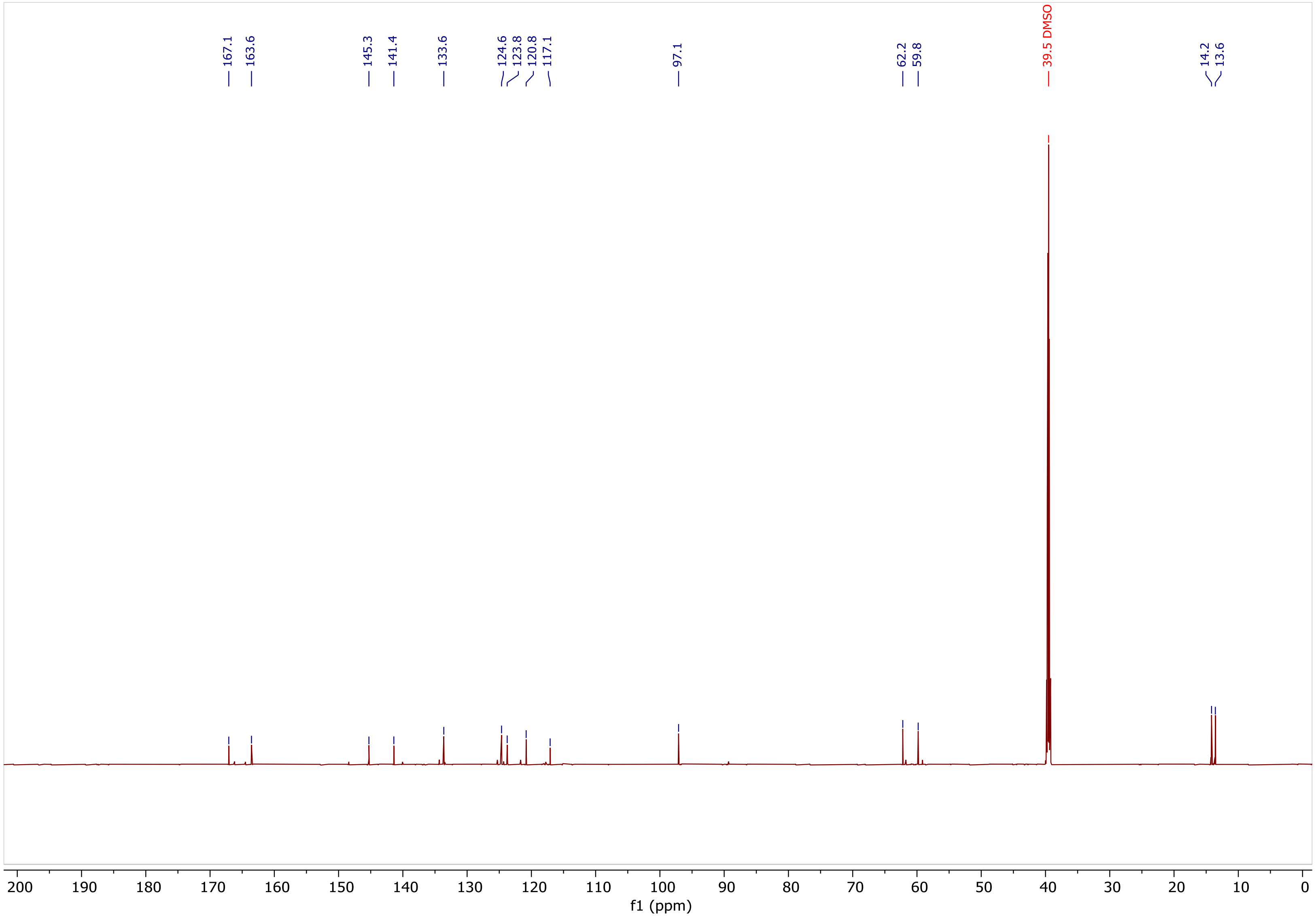

S19: NMR Data Table for Compound **9**[a]

Position	δ_H, mult. (*J* in Hz), int.	δ_C, mult.	COSY	HMBC
1	9.54, s, 1H			3, 7, 11, 16
2		128.1, C		
3		151.0, C		
4	7.21, d (2.1), 1H	114.6, CH		2, 6
5		116.0, C		
6	7.07, dd (8.5, 2.1), 1H	123.2, CH	7	2, 4
7	6.78, d (8.5), 1H	121.7, CH	6	3, 5
8		147.5, C		
9	5.28, s, 1H	92.3, CH		16
10		168.5, C		
12	4.13, q (7.1), 2H	59.7, CH_2	15	12, 15
13	1.22, t (7.1), 3H	14.2, CH_3	14	14
14		163.1, C		
16	4.15, q (7.1), 2H	61.9, CH_2	19	16
17	1.12, t, (7.1), 3H	13.6, CH_3	18	18
19	3.81, s, 3H	56.1, CH_3		3

[a] Recorded in DMSO-D_6, 800 MHz (^{1}H NMR) and 200 MHz (^{13}C NMR) at 25 °C; [w] Weak.

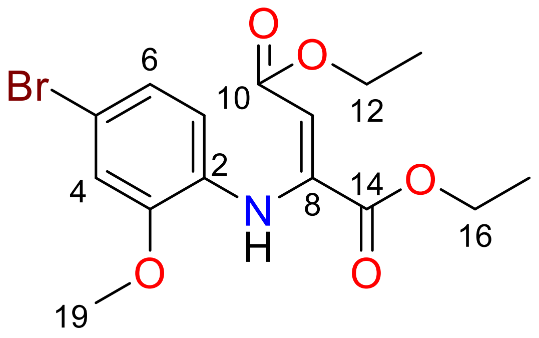

S20: ^{1}H NMR Spectrum of Compound **9** in DMSO-D_6

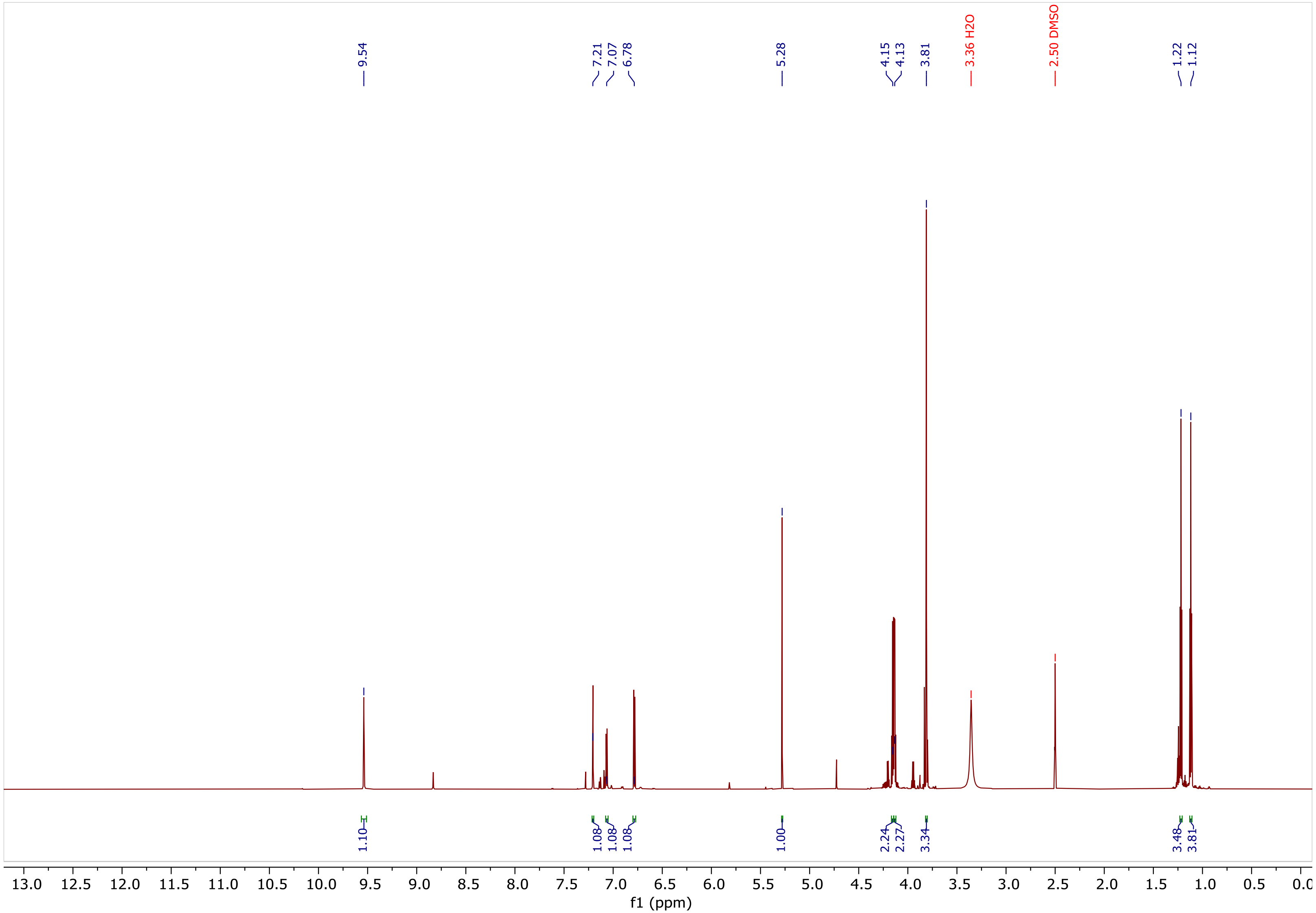

S21: ^{13}C NMR Spectrum of Compound **9** in DMSO-D$_6$

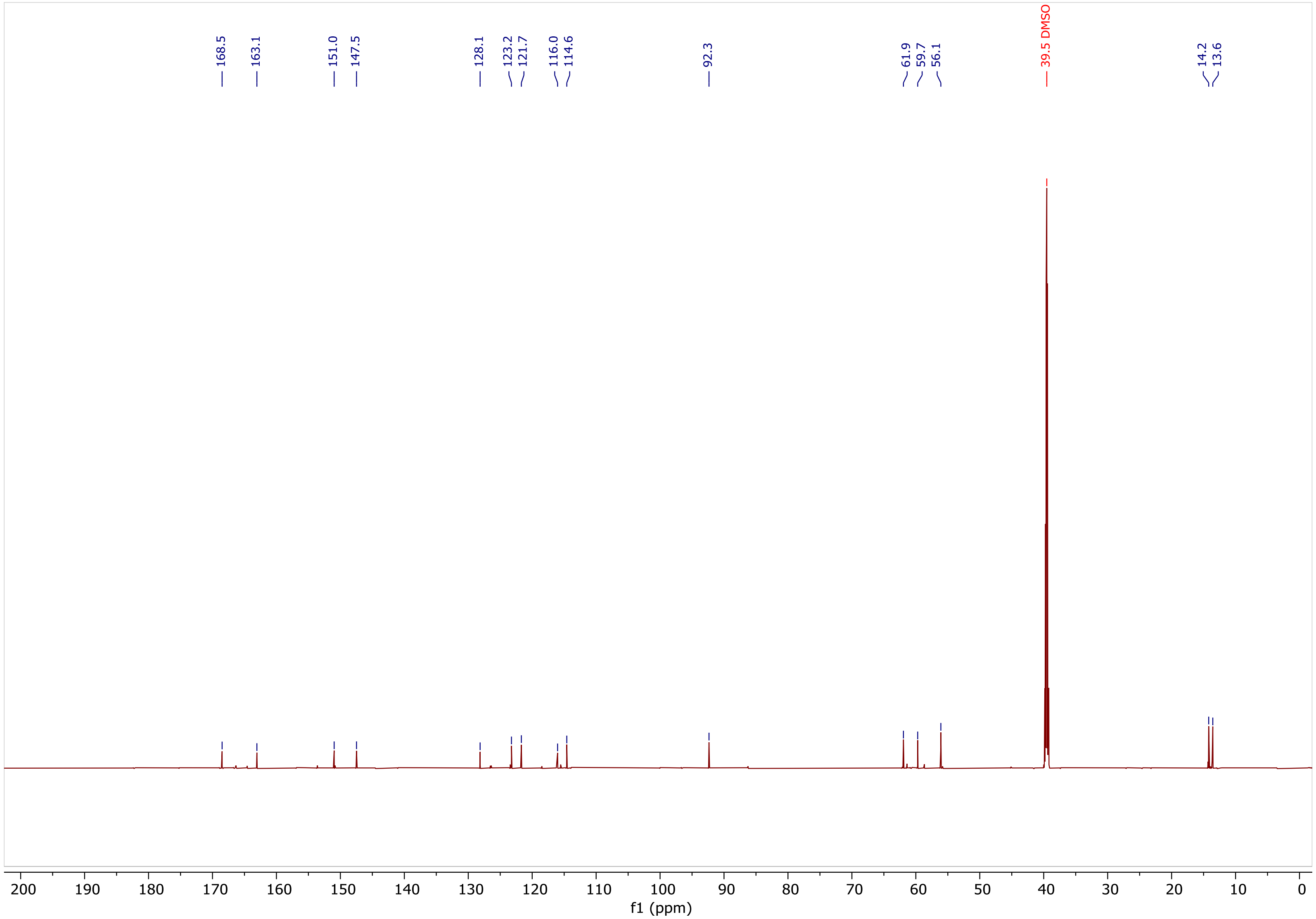

S22: NMR Data Table for Compound **10**[a]

Position	δ_H, mult. (*J* in Hz), int.	δ_C, mult.	COSY	HMBC
1	12.08, s, 1H			
2		139.7, C		
3	6.71, s, 1H	112.1, CH		2[w], 4a, 9
4		178.6, C		
4a		126.3, C		
5	8.10, d (8.6), 1H	128.3, CH	6	4, 7, 8a
6	7.58, dd (8.6, 1.8), 1H	128.2, CH	5, 8	4a, 8
7		127.5, C		
8	8.28, d (1.8), 1H	122.9, CH	6	4a, 6
8a		142.4, C		
9		163.3, C		
11	4.46, q (7.1), 2H	63.9, CH_2	12	9, 12
12	1.40, t (7.1), 3H	14.6, CH_3	11	11

[a] Recorded in DMF-D_7, 800 MHz (^{1}H NMR) and 200 MHz (^{13}C NMR) at 25 °C; [w] Weak.

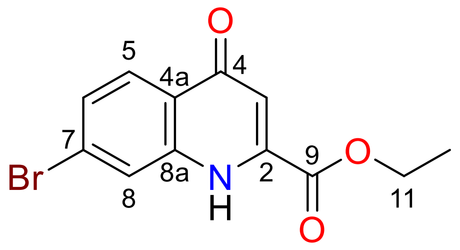

S23: ^{1}H NMR Spectrum of Compound **10** in DMF-D_7

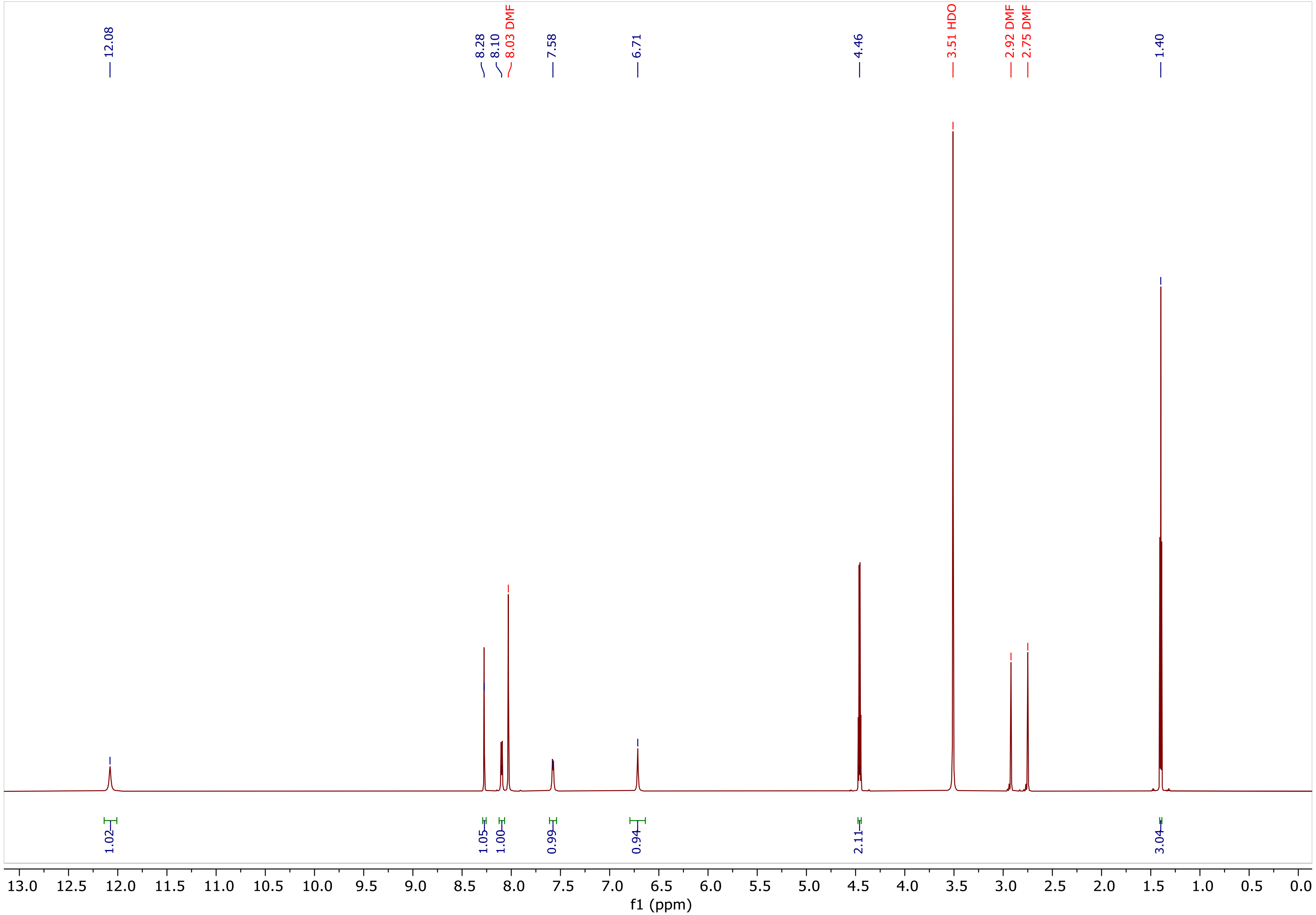

S24: ^{13}C NMR Spectrum of Compound **10** in DMF-D_7

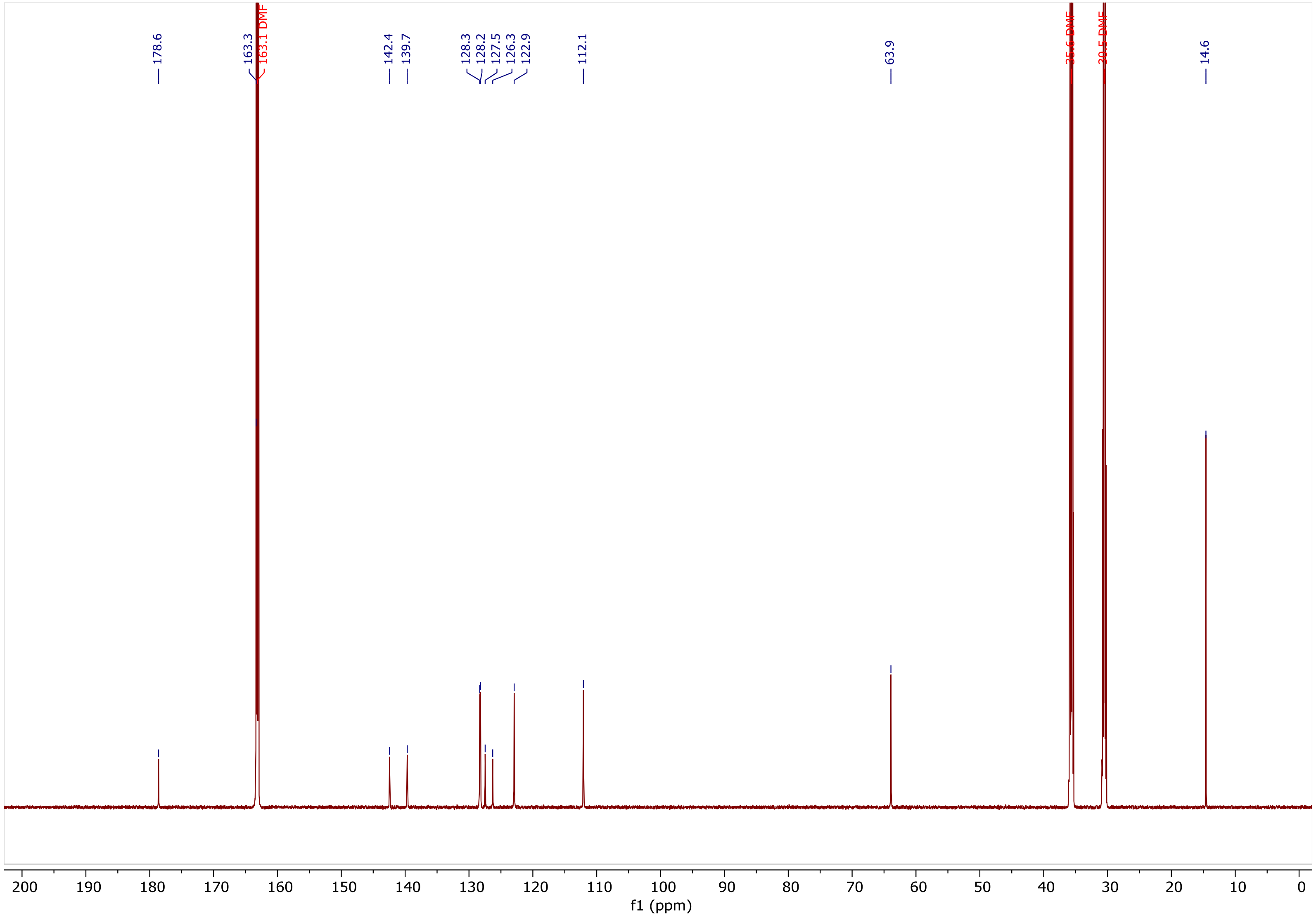

S25: NMR Data Table for Compound **11**[a]

Position	δ_H, mult. (J in Hz), int.	δ_C, mult.	COSY	HMBC
1	12.03, s, 1H			
2		138.2, C		
3	6.68, s, 1H	113.2, CH		2^w, 4a, 9
4		178.2, C		
4a		124.0, C		
5		120.7, C		
6	7.61, dd (7.3, 0.8), 1H	131.6, CH	7, 8^w	4a, 8
7	7.57, dd (8.3, 7.3), 1H	133.6, CH	5, 8	5, 8a
8	8.05, dd (8.3, 0.8), 1H	120.6, CH	6^w, 7	4a, 6
8a		144.1, C		
9		163.3, C		
11	4.45, q (7.1), 2H	63.8, CH_2	12	9, 12
12	1.39, t (7.1), 3H	14.6, CH_3	11	11

[a] Recorded in DMF-D_7, 800 MHz (^{1}H NMR) and 200 MHz (^{13}C NMR) at 25 °C; ^{w} Weak.

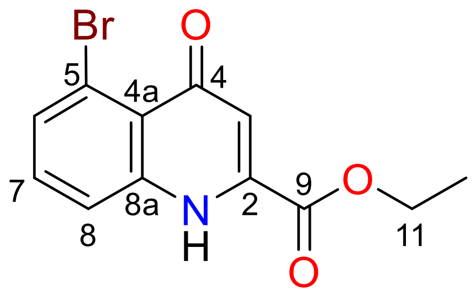

S26: ^{1}H NMR Spectrum of Compound **11** in DMF-D_7

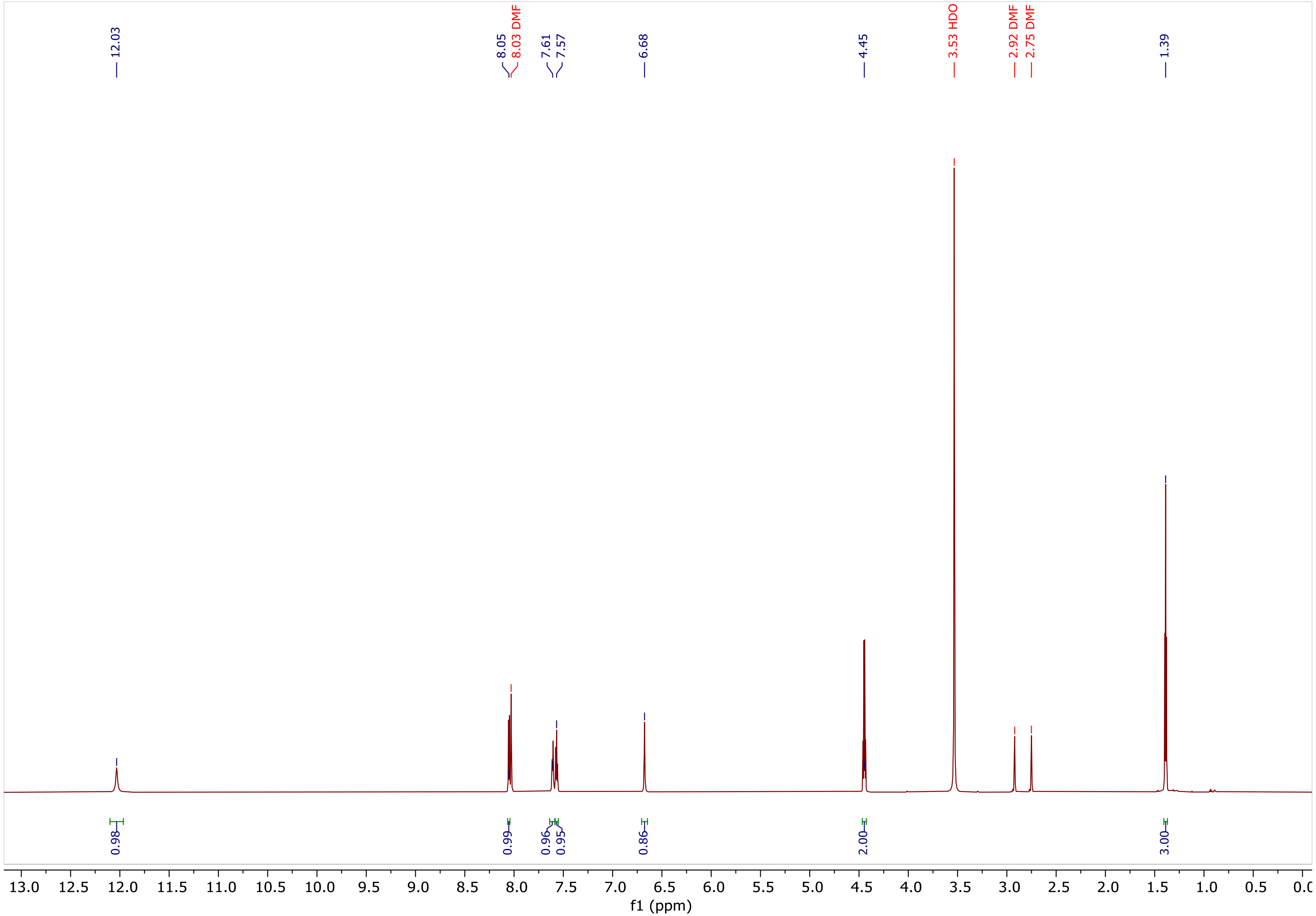

S27: ^{13}C NMR Spectrum of Compound **11** in DMF-D$_7$

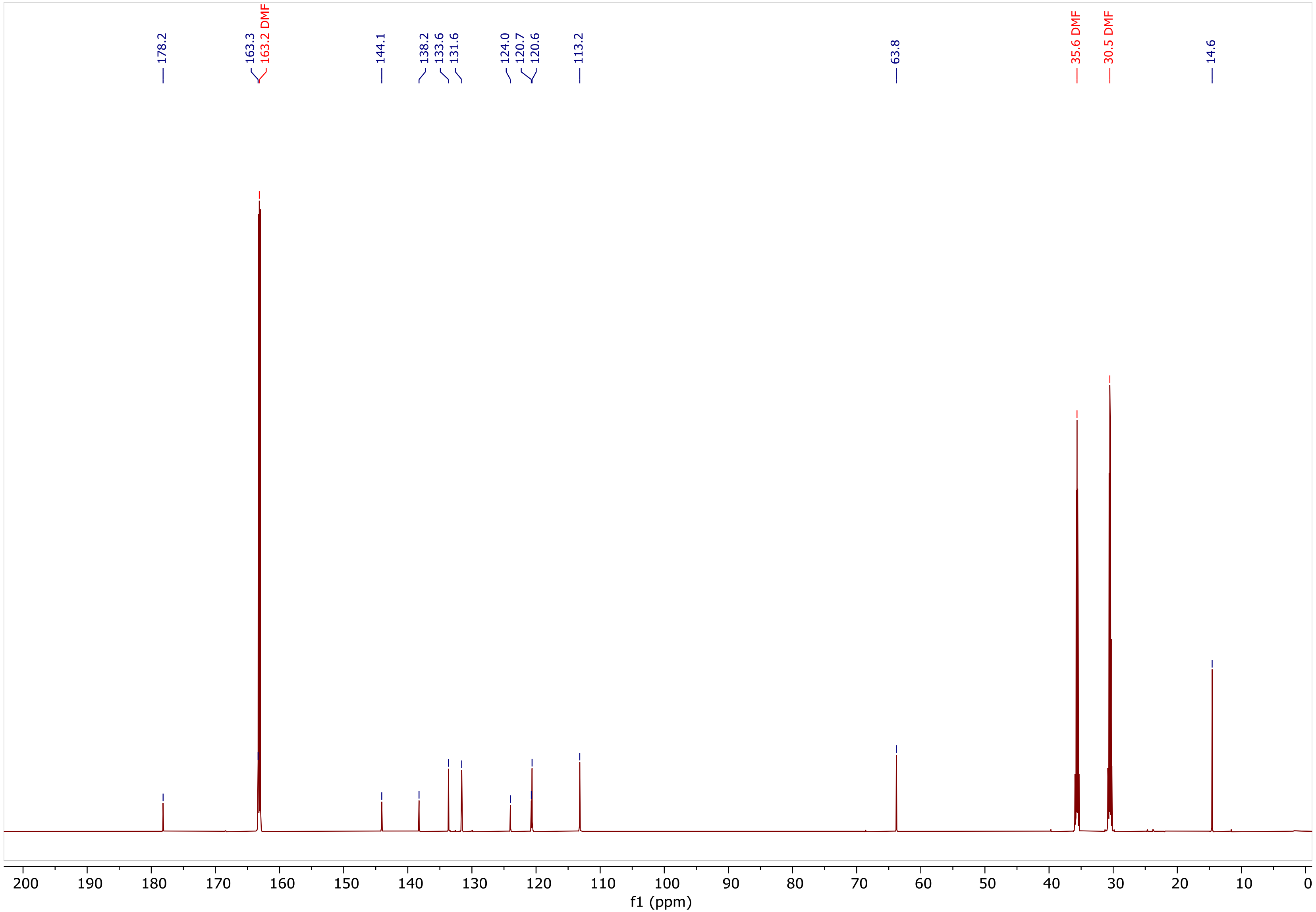

S28: NMR Data Table for Compound **12**[a]

Position	δ$_H$, mult. (*J* in Hz), int.	δ$_C$, mult.	COSY	HMBC
1	12.21, s, 1H			
2		140.1, C		
3	6.75, s, 1H	112.1, CH		
4		177.5, C		
4a		127.6, C		
5	8.38, s, 1H	130.7, CH		4, 7, 8a
6		120.3, C		
7		129.5, C		
8	8.46, s, 1H	125.8, CH		4a, 6
8a		141.2 C		
9		163.3, C		
11	4.47, q (7.2), 2H	64.0, CH_2	12	9, 12[w]
12	1.40, t (7.2), 3H	14.6, CH_3	11	11[w]

[a] Recorded in DMF-D_7, 800 MHz (^{1}H NMR) and 200 MHz (^{13}C NMR) at 25 °C with 2% Pyridine-D_5; [w] Weak.

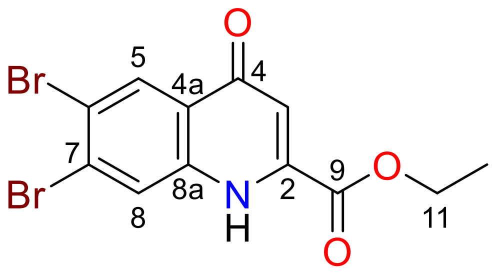

S29: ^{1}H NMR Spectrum of Compound **12** in DMF-D_7

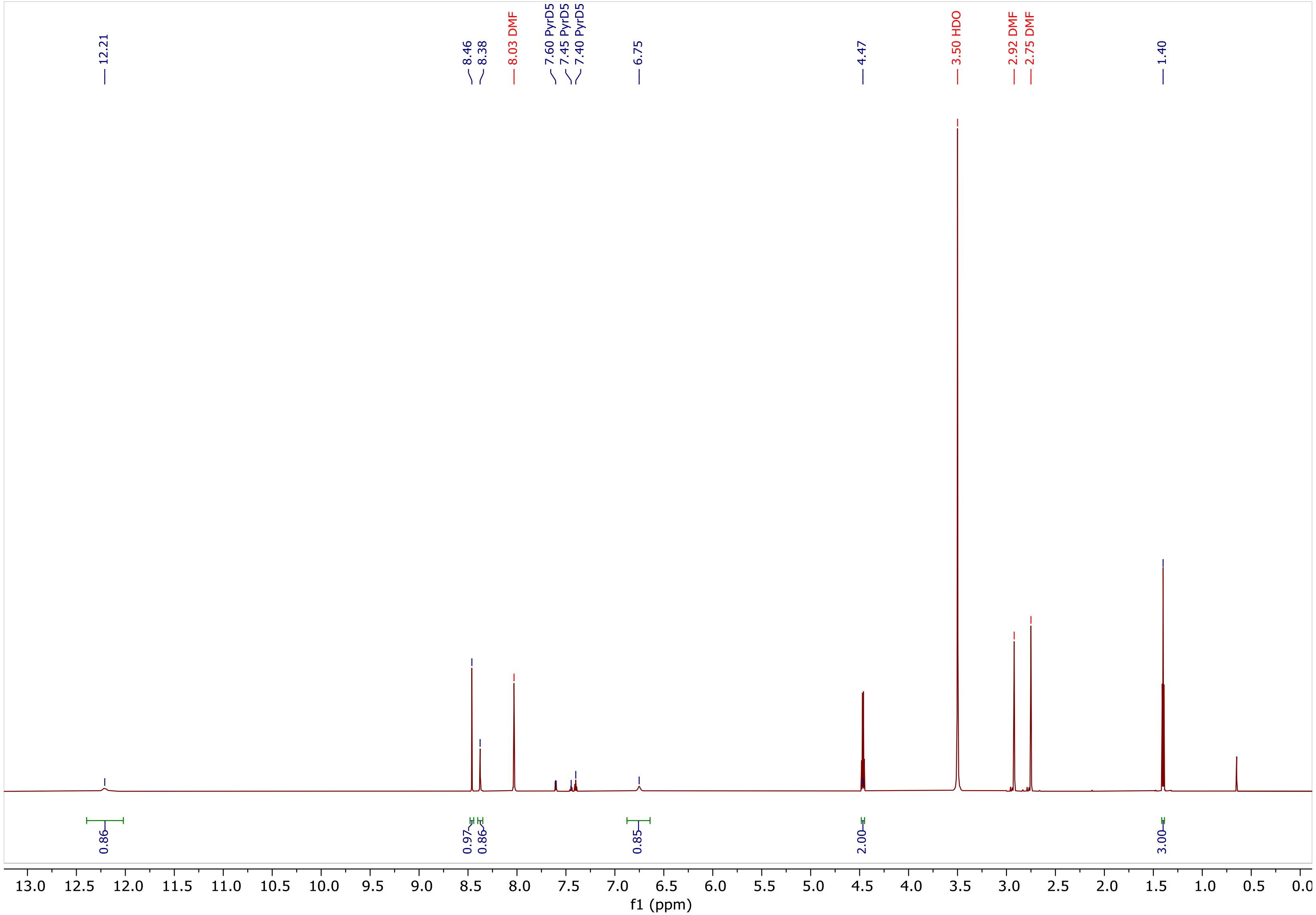

S30: ^{13}C NMR Spectrum of Compound **12** in DMF-D_7

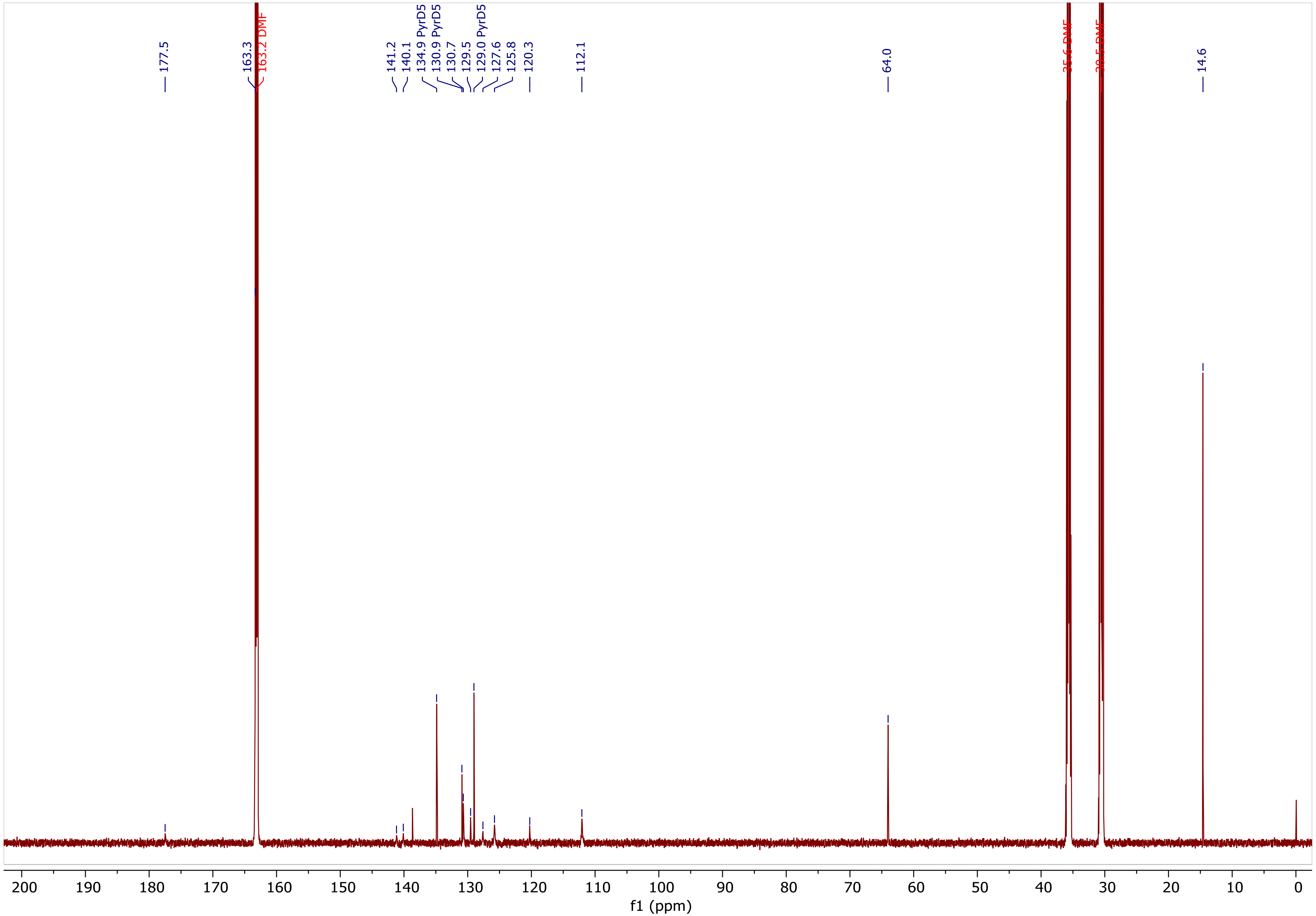

S31: NMR Data Table for Compound **13**[a]

Position	δ_H, mult. (*J* in Hz), int.	δ_C, mult.	COSY	HMBC
1	12.16, s, 1H			
2		138.2, C		
3	6.74, s, 1H	113.5, CH		
4		177.3, C		
4a		126.1, C		
5		122.7, C		
6		124.3, C		
7	8.04, d (9.0), 1H	137.4, CH	8	5, 8a
8	8.02, d (9.0), 1H	122.1, CH	7	4a, 6
8a		143.0 C		
9		163.3, C		
11	4.45, q (7.2), 2H	63.9, CH_2		9, 12[w]
12	1.39, t (7.2), 3H	14.6, CH_3		11

[a] Recorded in DMF-D_7, 800 MHz (^{1}H NMR) and 200 MHz (^{13}C NMR) at 25 °C; [w] Weak.

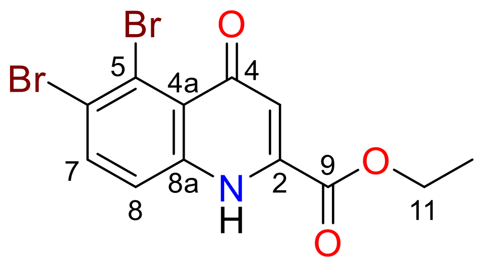

S32: ^{1}H NMR Spectrum of Compound **13** in DMF-D$_7$

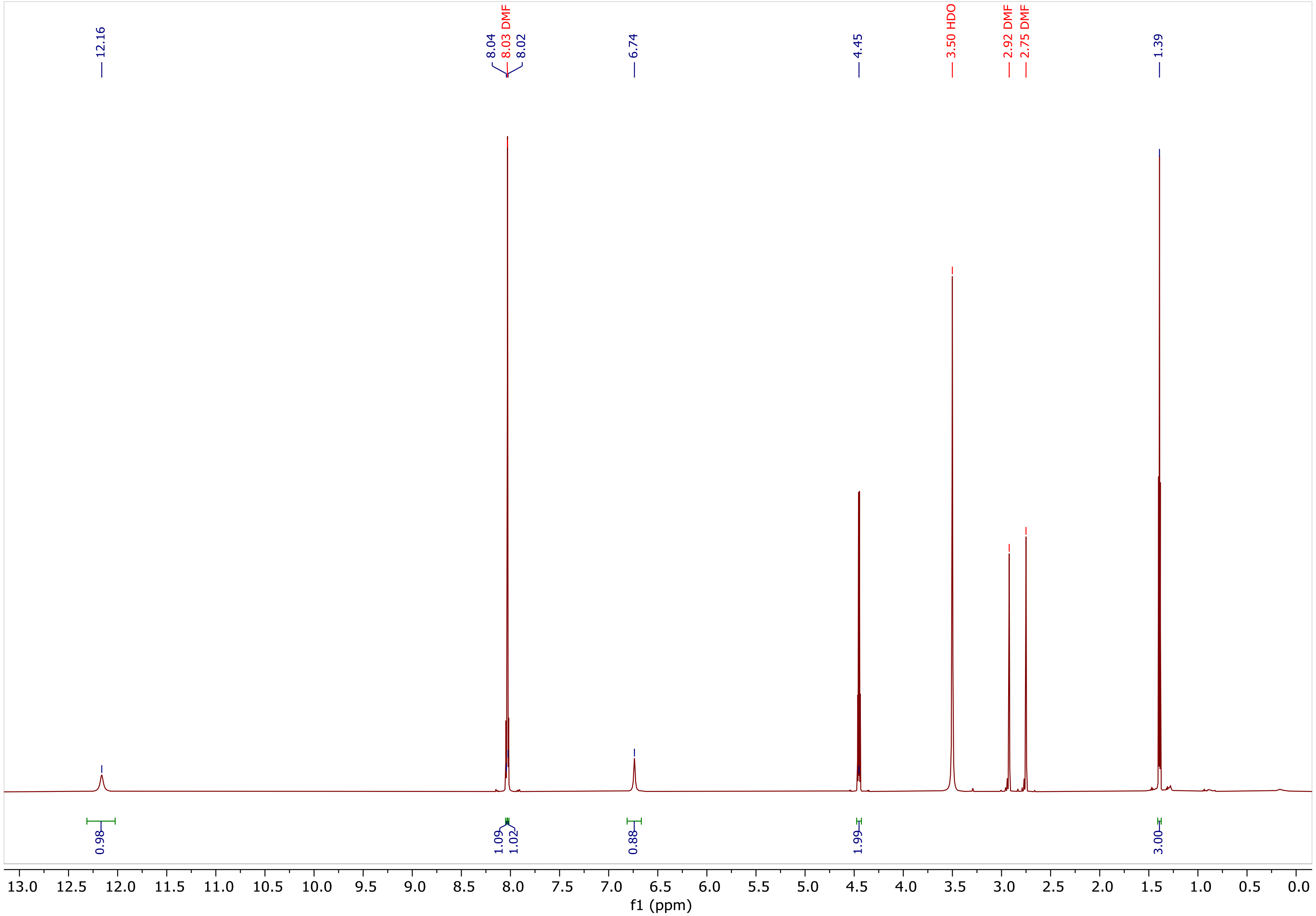

S33: ^{13}C NMR Spectrum of Compound **13** in DMF-D$_7$

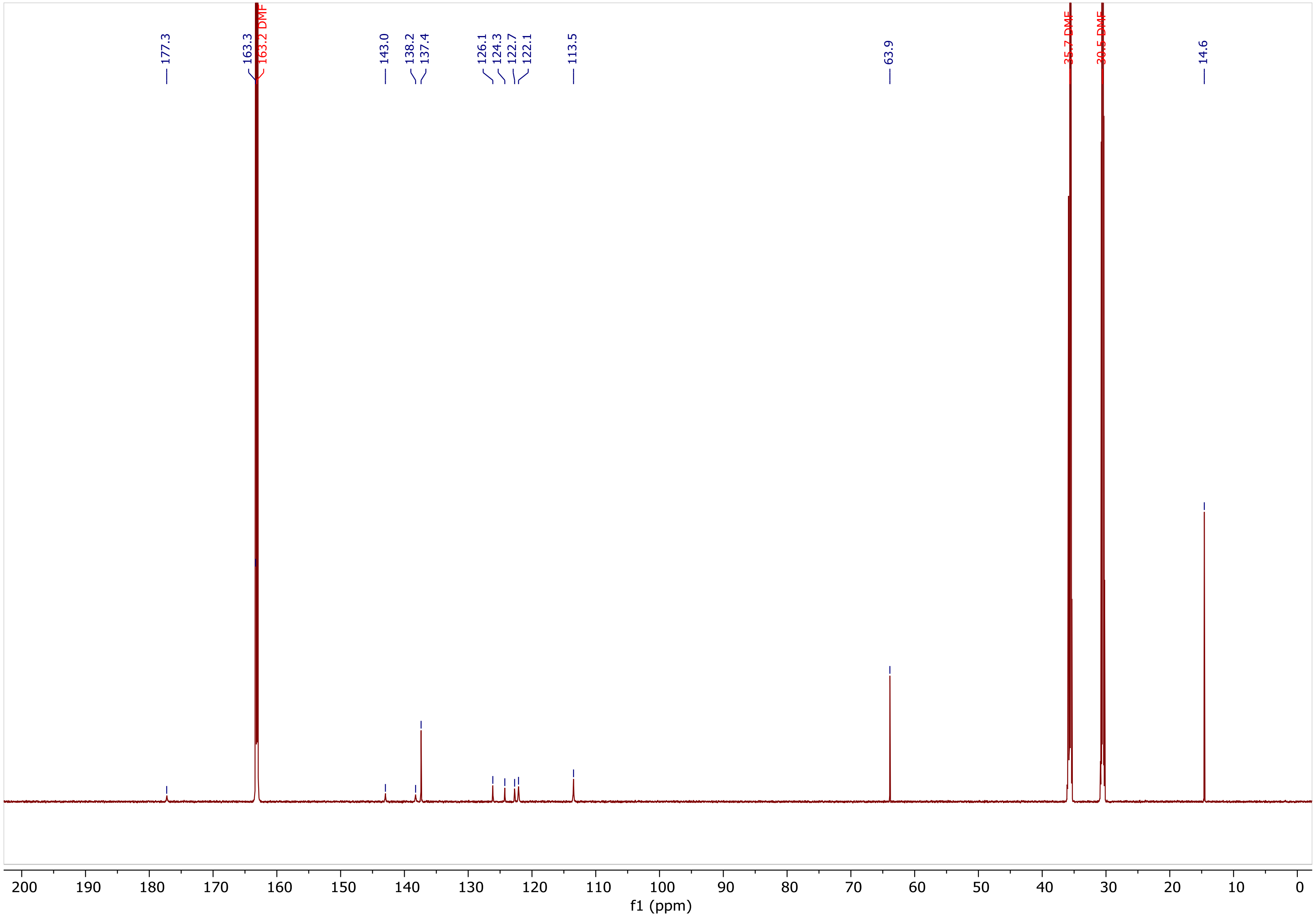

S34: NMR Data Table for Compound **14**[a]

Position	δ_H, mult. (*J* in Hz), int.	δ_C, mult.	COSY	HMBC
1	9.88, s, 1H			
2		138.6, C		
3	6.70, s, 1H	112.0, CH		
4		177.5, C		
4a		128.5, C		
5	7.80, s, 1H	119.5, CH		4, 7, 8a
6		118.3, C		
7	7.50, s, 1H	116.3, CH		5, 8a
8		151.1, C		
8a		130.8, C		
9		163.3, C		
11	4.48, q (7.1), 2H	64.2, CH_2	12	9, 12
12	1.42, t (7.1), 3H	14.6, CH_3	11	11
14	4.13, s, 3H	57.9, CH_3		8

[a] Recorded in DMF-D_7, 800 MHz (^{1}H NMR) and 200 MHz (^{13}C NMR) at 25 °C; [w] Weak.

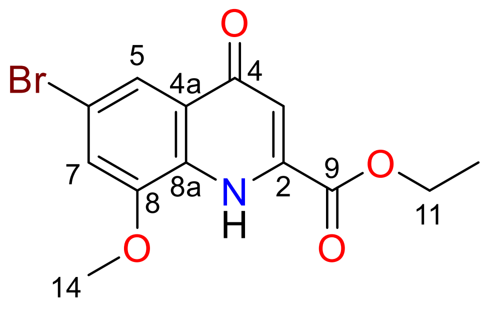

S35: ^{1}H NMR Spectrum of Compound **14** in DMF-D_7

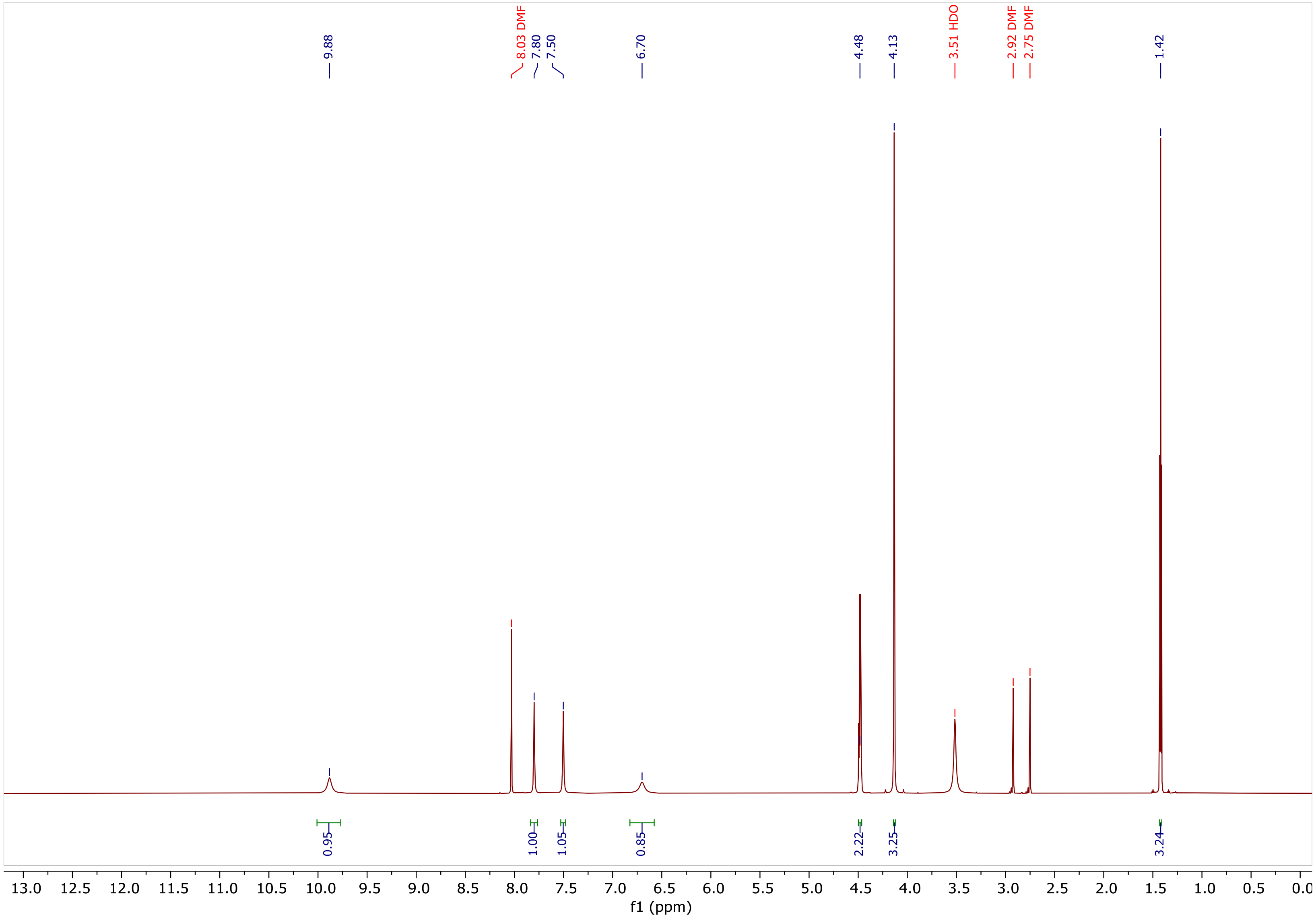

S36: ^{13}C NMR Spectrum of Compound **14** in DMF-D_7

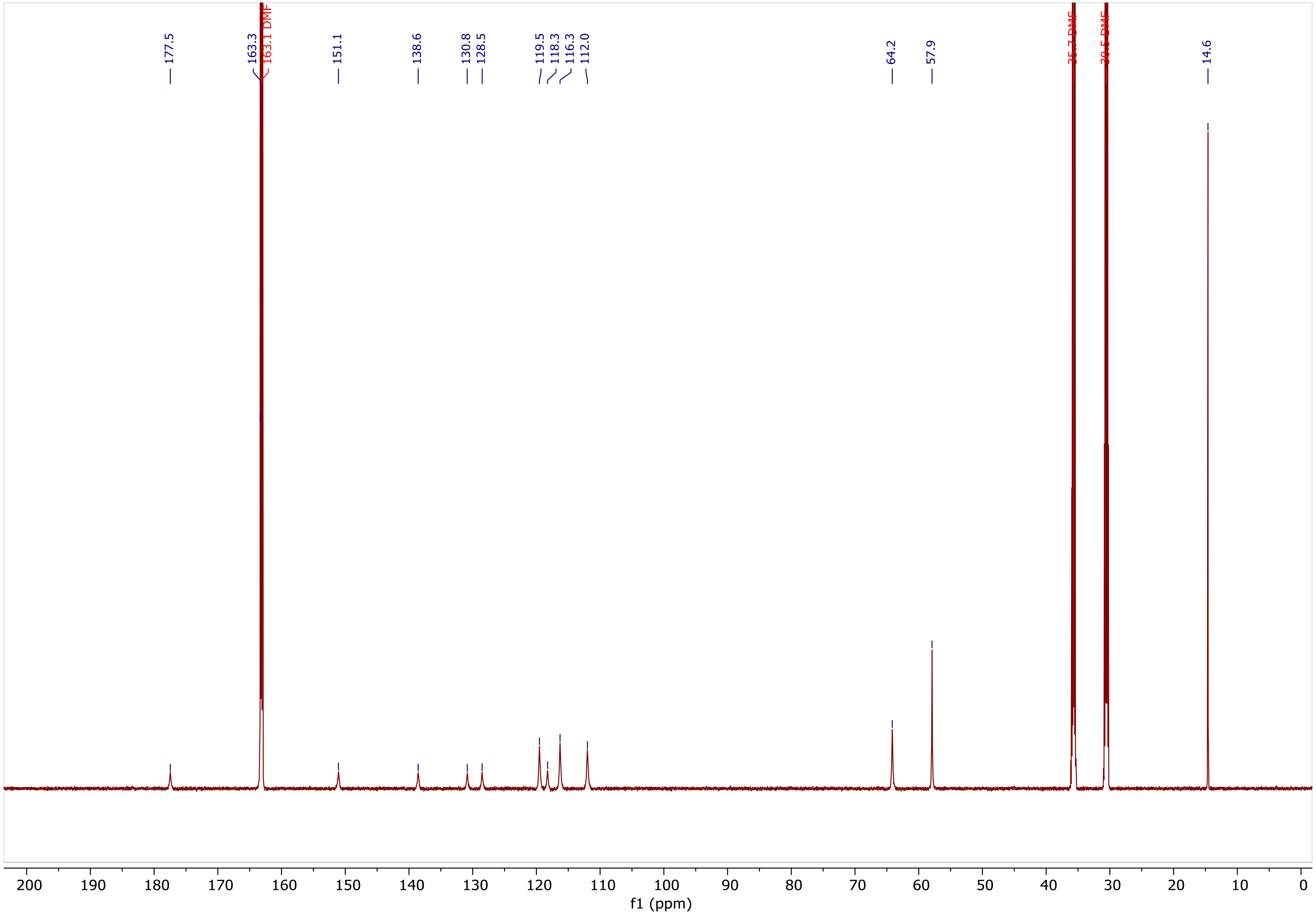

S37: NMR Data Table for Compound **15**[a]

Position	δH, mult. (*J* in Hz), int.	δC, mult.	COSY	HMBC
1	11.89, s, 1H			
2		139.4, C		
3	6.71, s, 1H	113.1, CH		2[w], 4a, 9
4		178.3, C		
4a		123.9, C		
5		120.7, C		
6	7.60, dd (7.6, 1.1), 1H	131.5, CH	7, 8[w]	4a, 8
7	7.56, dd (8.3, 7.6), 1H	133.5, CH	6, 8	5, 8a
8	8.10, dd (8.3, 1.1), 1H	120.8, CH	6[w], 7	4a, 6, 8a[w]
8a		144.2 C		
2-COOH		164.6, C		

[a] Recorded in DMF-D7, 800 MHz (^{1}H NMR) and 200 MHz (^{13}C NMR) at 25 °C with 2% MeOD4;
[w] Weak.

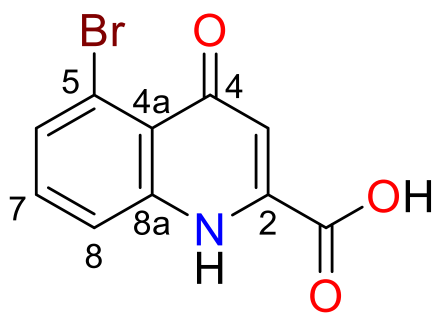

S38: ^{1}H NMR Spectrum of Compound **15** in DMF-D_7

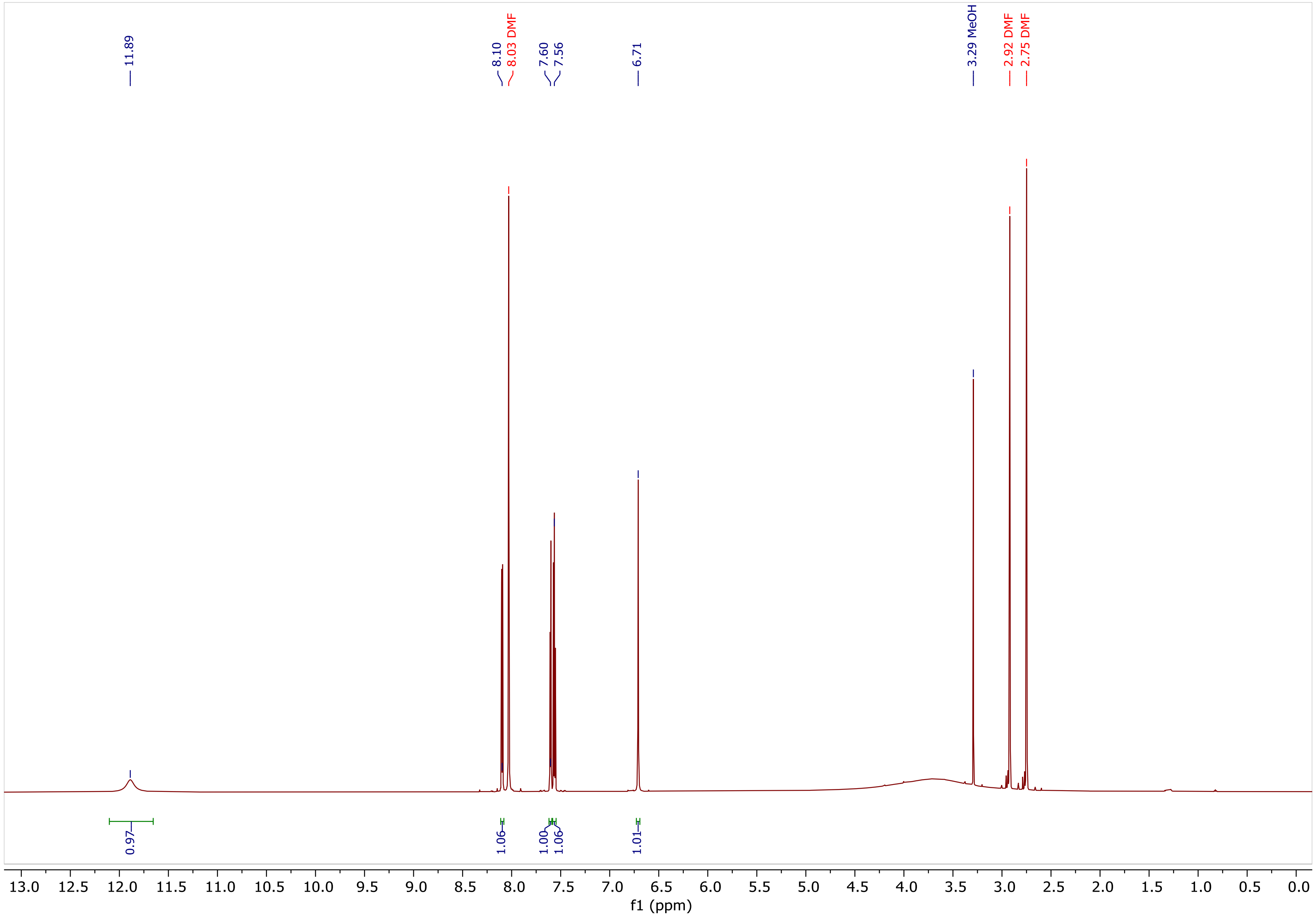

S39: ^{13}C NMR Spectrum of Compound **15** in DMF-D_7

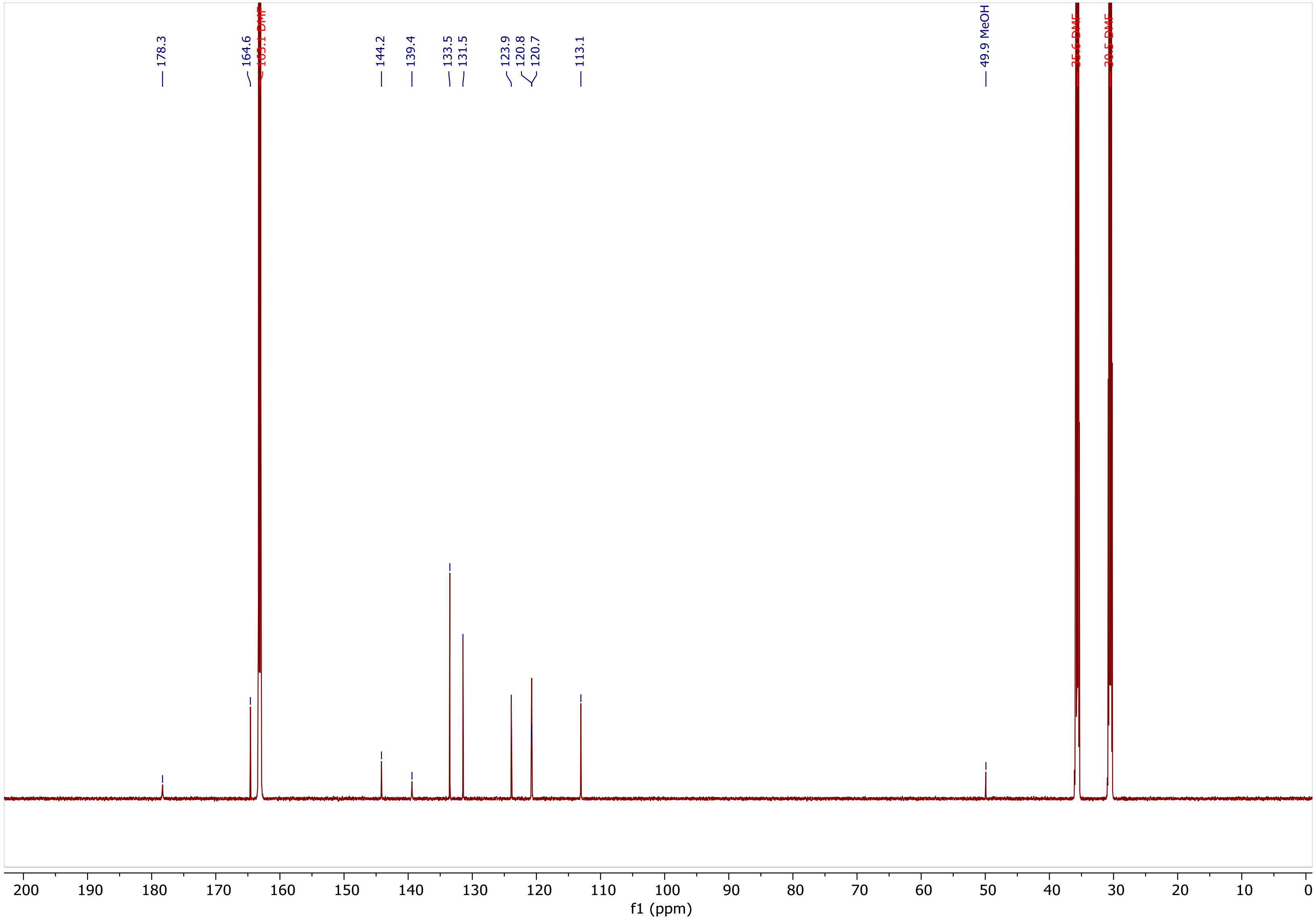

S40: NMR Data Table for Compound **16**[a]

Position	δ_H, mult. (*J* in Hz), int.	δ_C, mult.	COSY	HMBC
1	N.D.			
2		146.9, C		
3	6.74, s, 1H	111.2, CH		4a, 9
4		178.2, C		
4a		125.7, C		
5		122.7, C		
6		122.8, C		
7	7.99, d (9.0), 1H	136.2, CH	8	5, 8a
8	8.09, d (9.0), 1H	122.1, CH	7	4a, 6
8a		142.5 C		
2-COOH		164.4, C		

[a] Recorded in DMF-D_7, 800 MHz (^{1}H NMR) and 200 MHz (^{13}C NMR) at 25 °C with 2% $MeOD_4$; N.D. Not Detected; [w] Weak.

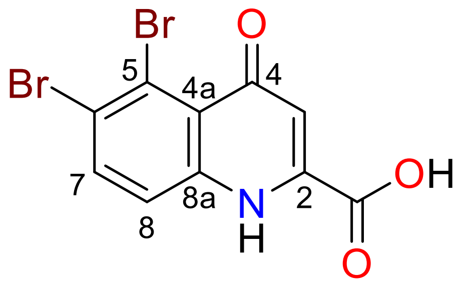

S41: ^{1}H NMR Spectrum of Compound **16** in DMF-D_7

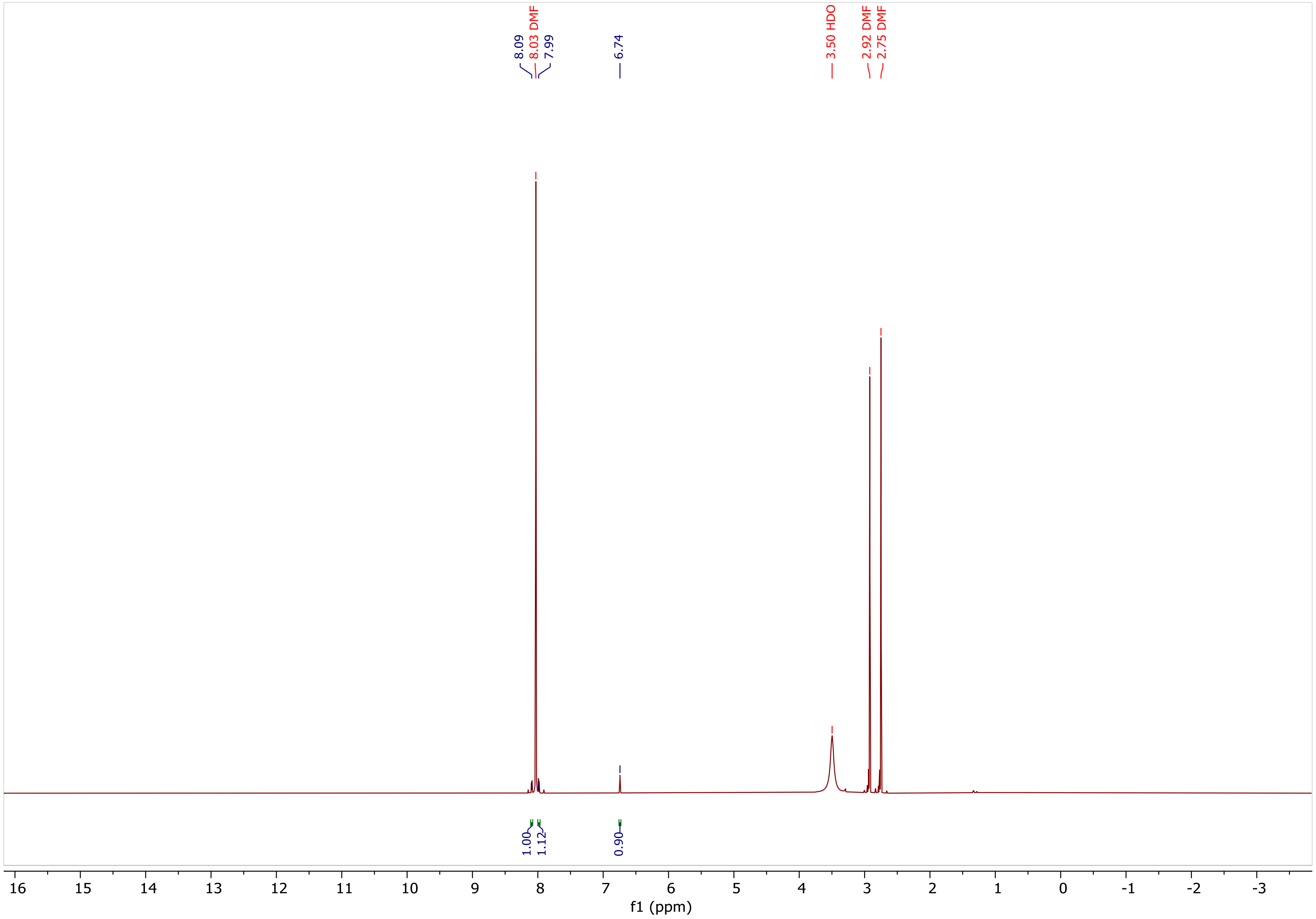

S42: ^{13}C NMR Spectrum of Compound **16** in DMF-D_7

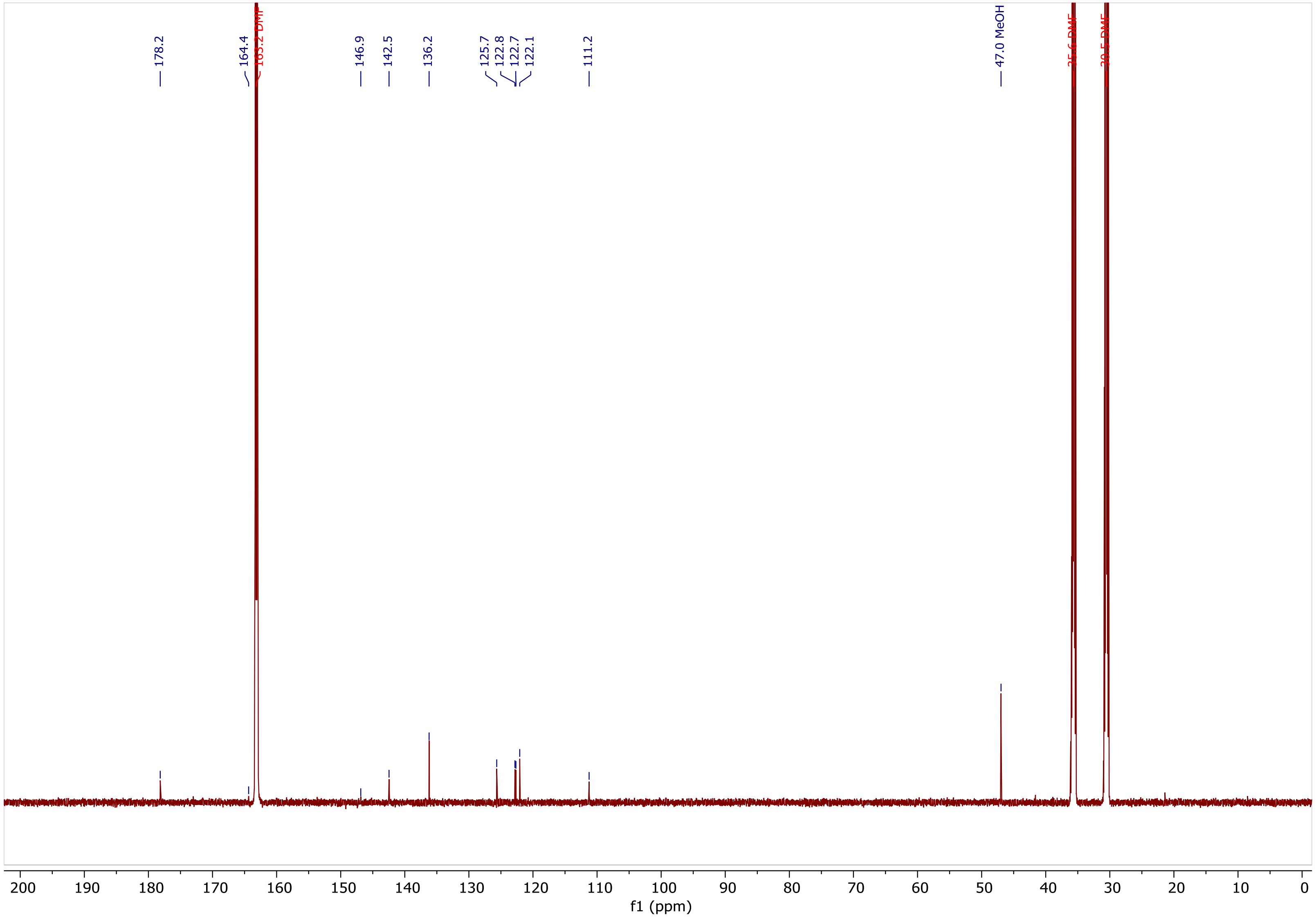

S43: Biological Data

Table 1: Biological data for activity of the caelestines A–C (**1**–**3**) and related quinolones (including the novel quinolones **12**–**14** and **16**) against *P. falciparum* 3D7 and Dd2, and the non-tumorigenic cell line HEK293.

**Compound**	***Pf* 3D7[a] IC_{50} ± SD µM**	***Pf* Dd2[b] IC_{50} ± SD µM**	**HEK293 IC_{50} ± SD µM**	**Selectivity Index[c] (3D7)**	**Selectivity Index[c] (Dd2)**
**1**	-	-	-	-	-
**2**	4.6 ± 0.8	4.4 ± 0.4	-	> 9	> 9
**3**	-	-	-	-	-
**10**	-	-	-	-	-
**11**	-	-	-	-	-
**12**	34 ± 29[d]	52 ± 15[d]	-	-	-
**13**	-	-	-	-	-
**14**	-	-	-	-	-
**15**	-	-	-	-	-
**16**	-	-	-	-	-
**Reference Compounds**	***Pf* 3D7[a] IC_{50} ± SD µM**	***Pf* Dd2[b] IC_{50} ± SD µM**	**HEK293 IC_{50} ± SD µM**	**Selectivity Index[c] (3D7)**	**Selectivity Index[c] (Dd2)**
PMY	0.047 ± 0.011	0.040 ± 0.009	0.40 ± 0.11	9	10
CQ	0.014 ± 0.004	0.18 ± 0.05	-	-	-
ATN	0.003 ± 0.001	0.004 ± 0.001	-	-	-

[a] *Pf* 3D7 = *P. falciparum* 3D7 (chloroquine-sensitive strain); [b] *Pf* Dd2 = *P. falciparum* Dd2 (chloroquine-resistant strain); [c] Selectivity index (fold difference) with respect to compound activity against HEK293 *i.e.* $IC_{50}^{(HEK293)}/IC_{50}^{(Pf3D7)}$; [d] Mean % inhibition ± SD at assay top dose (40 µM). SD = standard deviation, PMY = puromycin, CQ = chloroquine, ATN = artesunate. HEK293 experiments were performed as *N* = 1, triplicate point, including reference compound, puromycin. Antimalarial experiments were *N* = 3, single point. Reference compounds were used as in-plate controls (*N* = 3, duplicate point) for all antimalarial assay plates, and the data presented for those reference compounds is indicative of six replicates.

S44: Chromatography Studies

Quinolone synthesis yielded semipure **14** and isomeric mixtures of **10** and **11** from **7**, and mixtures of **12** and **13** from **8**. All quinolones, including the ethyl carboxylates (**10**–**14**) and carboxylic acids (**1**–**3** and **15**–**16**) exhibited extremely aberrant chromatography during reversed-phase semi-preparative HPLC (RP-HPLC) under a range of conditions (**Table 2**). Compound peaks showed a pattern of broad and bulk elution followed by severe tailing that was prolonged for several column volumes and prevented the efficient recovery of pure isomer (**Figure 1A**). This elution pattern occurred regardless of whether the samples were pre-adsorbed to functionalised silica in a guard cartridge for in-line loading, or were injected as a liquid, and occurred for pure compounds (which were isolated after an effective method was developed), as well as for semipure isomeric mixtures. The elution pattern was also preserved across different HPLC systems but was absent in analytical UHPLC separations. The aberrant chromatography observed continued to occur under different solvent systems, column packings and at varying pH (**Table 2**).

Table 2: Conditions trialled for separation of 2-carboxy-4-quinolones synthesised in these studies. FA = formic acid, GAA = glacial acetic acid, TEA = triethylamine, TFA = trifluoroacetic acid.

**Acidic Conditions**					
Organic Phase	Column	Acid Additive	Counter Salt	Measured pH	Separation
		0.1% FA	-	2.7	No
	XBridge™ C_{18}	0.1%TFA	-	1.9	No
		None	-	7.0	No
		0.1% FA	-	2.7	No
MeCN	Betasil C_{18}	0.1%TFA	-	1.9	No
		None	-	7.0	No
		0.1% FA	-	2.7	No
	Betasil Phenyl	0.1%TFA	-	1.9	No
		None	-	7.0	No
		0.1% FA	-	2.7	No
	XBridge™ C_{18}	0.1%TFA	-	1.9	No
		None	-	7.0	No
		0.1% FA	-	2.7	No
MeOH	Betasil C_{18}	0.1%TFA	-	1.9	No
		None	-	7.0	No
		0.1% FA	-	2.7	No
	Betasil Phenyl	0.1%TFA	-	1.9	No
		None	-	7.0	No
**Basic Conditions**					
Organic Phase	Column	Base Additive	Counter Salt	Adjusted pH	Separation
		0.1% TEA	None	-	No
		0.3%TEA	None	-	No
			GAA 0.3%	5.0	No
MeCN	XBridge™ C_{18}		GAA 0.3%	7.0	No
		0.3% TEA	GAA 0.3%	9.0	No
			GAA 0.3%	11.0	No
			GAA 0.3%	12.0	Yes
		0.1% TEA	None	-	No
		0.3%TEA	None	-	No
			GAA 0.3%	5.0	No
MeOH	XBridge™ C_{18}		GAA 0.3%	7.0	No
		0.3% TEA	GAA 0.3%	9.0	No
			GAA 0.3%	11.0	No
			GAA 0.3%	12.0	Yes

Amongst the conditions tested, the only effective separation using a standard HPLC system (**Figure 1B**) was accomplished at pH 12.0 with 0.3% v/v triethylamine (TEA) in both H_2O and the organic phase (MeCN or MeOH), with or without acetate as counter salt, so long as the pH remained highly alkaline. This required the use of a specialty bonded column (bridged ethylene hybrid or BEH, XBridge™ C_{18}) which was tolerant of alkaline conditions but was not a practical solution given that the pH required, pH 12.0, was at the limit of the tolerance of this column, and of similar commercially available columns. The alkali conditions also hydrolysed any ethyl carboxylates injected (**10**–**14**). In light of this, it appeared unlikely that solvent, salt or additive systems commonly employed for semi-preparative RP-HPLC method development would lead to separation in the absence of highly alkaline conditions.

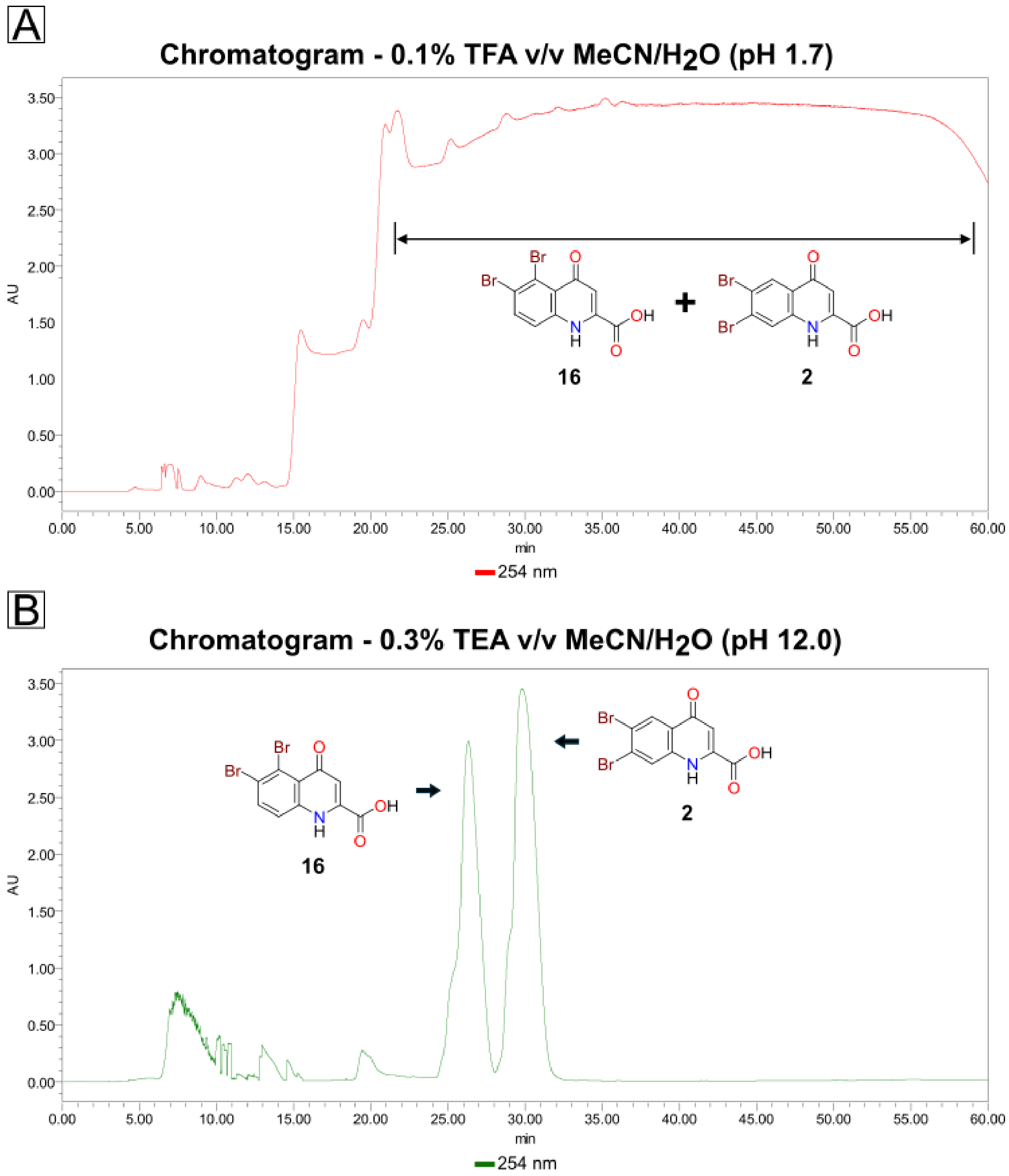

Figure 1: Illustrative comparison of chromatographic separation (RP-HPLC) of semipure isomeric mixture of **2** and **16**, when conducted under different conditions (XBridge[TM] BEH C_{18} column). **Panel A:** aberrant chromatography with extreme tailing observed under acidic conditions. **Panel B:** Entirely resolved separation of **2** and **16** under highly alkaline conditions.

UHPLC gave nominal chromatography (**Figure 2A**) and appeared to effectively separate isomeric product mixtures under acidic conditions (Betasil C_{18} 2.6 μM, 0.1% v/v formic acid with MeOH/H_2O). Although semi-preparative scale UHPLC is uncommon and was not at hand, another system, the Waters' LC Prep AutoPurification System was available in these studies. This autopurification system features a dual autoinjector/fraction collector with flow-split analytical capabilities and RP-HPLC component, but the RP-HPLC is conducted at a high flow rate (≥ 20 mL/min). Despite this high flow rate, the autopurification system operates at back-pressures typical of RP-HPLC (2500–5000 PSI). Test injections of semipure mixed isomers were conducted using the autopurification system, and despite ubiquitous peak broadening, both isomeric mixtures were very well resolved (**Figure 2B**), and the problematic extreme tailing was absent for all quinolones injected onto the system, which were subsequently isolated in very high purity (≥ 95%), even under acidic conditions (MeOH/H_2O with 0.1% v/v TFA, XBridge^{TM} C_{18}). These data suggest that the column interactions responsible for the extreme tailing observed are not mediated by the protonation state of the carboxylic acid (where applicable), or the protonation state of the quinoline nitrogen, or even tautomerisation exhibited by 2C4Qs, given that complete resolution of tailing was achieved over a large pH range, namely with 0.1% v/v TFA (pH 1.9) and on a UHPLC system with 0.01% v/v formic acid (pH 3.4), and also with 0.3% v/v TEA (pH 12.0). It is acknowledged that this chromatographic system is uncommon, as are semi-preparative UHPLC systems, but it appears that both UHPLC and high flow rate systems mitigate those interactions between 2C4Qs and reversed-phase columns which produce the extreme tailing.

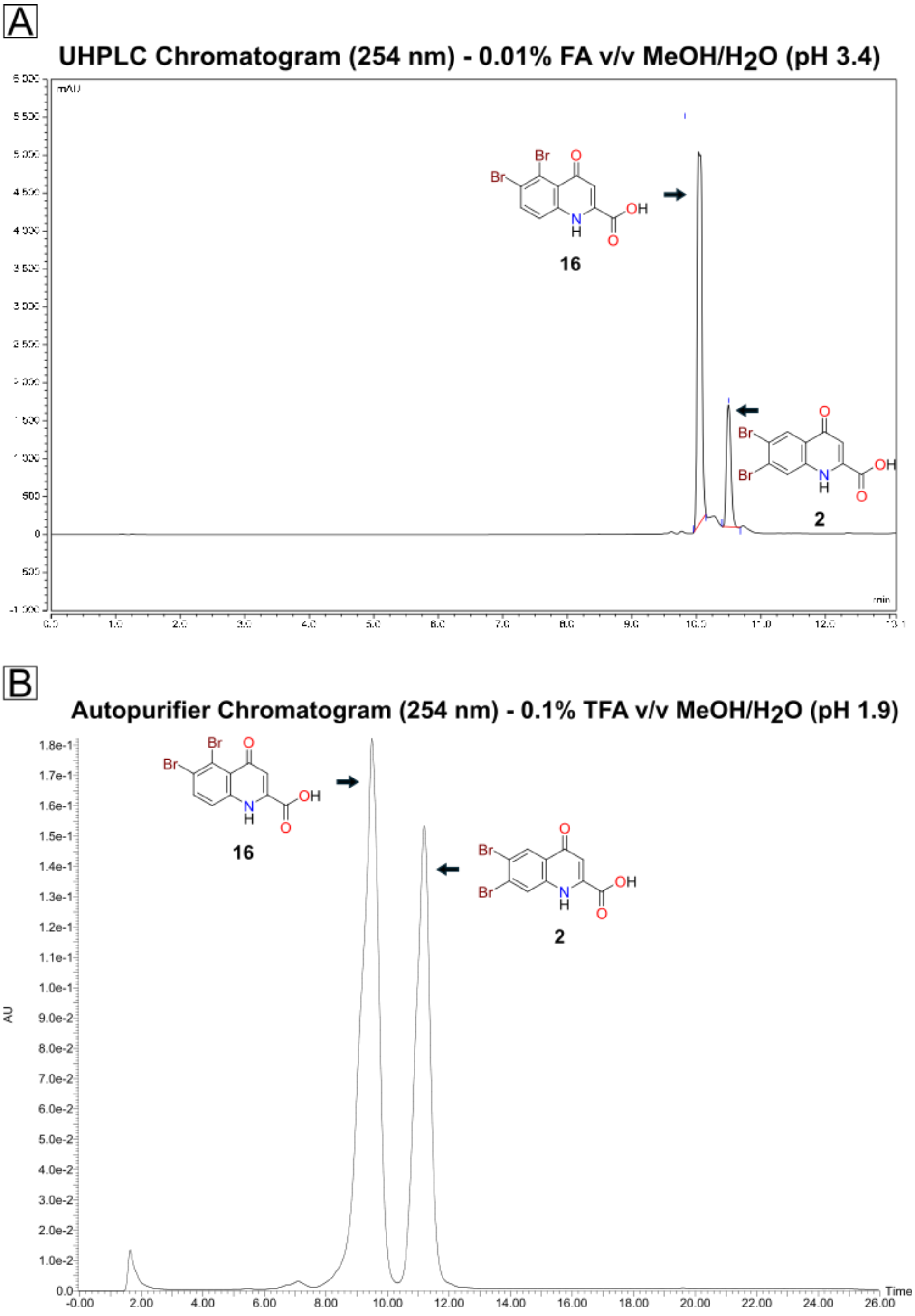

Figure 2: Illustrative example of effective separation of semipure mixed isomers of **2** and **16** under acidic conditions. **Panel A:** UHPLC separation of **2** and **16**. **Panel B:** Separation of semipure mixed isomer of **2** and **16** using the Waters' autopurification system under standard acidic conditions (XBridge™ C_{18} column, MeOH/H_2O with 0.1% v/v TFA).
